# Functional and Structural Mimicry of Cellular Protein Kinase A Anchoring Proteins by a Viral Oncoprotein

**DOI:** 10.1371/journal.ppat.1005621

**Published:** 2016-05-03

**Authors:** Cason R. King, Michael J. Cohen, Gregory J. Fonseca, Brennan S. Dirk, Jimmy D. Dikeakos, Joe S. Mymryk

**Affiliations:** 1 Department of Microbiology & Immunology, University of Western Ontario, London, Ontario, Canada; 2 Department of Oncology, University of Western Ontario, London, Ontario, Canada; 3 London Regional Cancer Program and Lawson Health Research Institute, London, Ontario, Canada; Stony Brook University, UNITED STATES

## Abstract

The oncoproteins of the small DNA tumor viruses interact with a plethora of cellular regulators to commandeer control of the infected cell. During infection, adenovirus E1A deregulates cAMP signalling and repurposes it for activation of viral gene expression. We show that E1A structurally and functionally mimics a cellular A-kinase anchoring protein (AKAP). E1A interacts with and relocalizes protein kinase A (PKA) to the nucleus, likely to virus replication centres, via an interaction with the regulatory subunits of PKA. Binding to PKA requires the N-terminus of E1A, which bears striking similarity to the amphipathic α-helical domain present in cellular AKAPs. E1A also targets the same docking-dimerization domain of PKA normally bound by cellular AKAPs. In addition, the AKAP like motif within E1A could restore PKA interaction to a cellular AKAP in which its normal interaction motif was deleted. During infection, E1A successfully competes with endogenous cellular AKAPs for PKA interaction. E1A’s role as a viral AKAP contributes to viral transcription, protein expression and progeny production. These data establish HAdV E1A as the first known viral AKAP. This represents a unique example of viral subversion of a crucial cellular regulatory pathway via structural mimicry of the PKA interaction domain of cellular AKAPs.

## Author Summary

Studies of human adenovirus (HAdV), a small DNA tumor virus, illustrate the profound impact of viral proteins on multiple host functions. The multifunctional E1A proteins of HAdV are particularly adept at targeting key cellular regulators. Mechanistically, E1A alters or inhibits the normal function of the cellular proteins that it targets, and also establishes new connections in the cellular protein interaction network. Through these interactions, E1A creates a cellular milieu more conducive for replication. Here we show that HAdV E1A mimics cellular A-kinase anchoring proteins (AKAPs) in both appearance and function. We found that the protein kinase A (PKA) regulatory subunits are conserved targets of most HAdV E1A species. Structural modeling and a docking analysis predict a remarkable similarity between the binding of E1A and cellular AKAPs to PKA, which was confirmed experimentally. In addition, we observed E1A-mediated relocalization of PKA subunits and competition between E1A and cellular AKAPs during infection that contribute to HAdV gene expression and overall viral replication. Together, our studies identify E1A as the first known viral AKAP, and reveal a unique example of viral subversion of the PKA pathway via structural mimicry.

## Introduction

As obligate intracellular parasites, all viruses are critically dependent upon the host cell. Intensive selective pressure, rapid replicative cycle times and severe restrictions on viral genome size combine to drive virus evolution. As a consequence, viral regulatory proteins have been relentlessly forged into exquisitely sophisticated instruments that functionally reprogram the infected cell [1]. Studies of human adenovirus (HAdV), a small DNA tumor virus, illustrate the profound impact of viral proteins on multiple host functions to maximize viral propagation [2–7].

The multifunctional E1A proteins of HAdV are particularly adept at targeting key cellular regulators. Through these interactions, E1A creates a cellular milieu more conducive for replication. Indeed, E1A enhances cell cycle entry, subverts innate immunity and intensively reprograms the cellular gene expression program [5,6,8]. The modular E1A proteins are dense with short linear sequence motifs that bind to and alter the activity of dozens of critical cellular proteins [9,10]. Many of the interaction motifs in E1A are functional mimics of highly similar sequences present in cellular regulatory proteins. Thus, viral evolution has converged to generate specific high affinity protein interaction surfaces that perturb cell regulation by competing with endogenous targets.

Cellular compartmentalisation of proteins is a widespread cellular mechanism that ensures the interaction of signalling molecules with a localized subset of appropriate effector proteins. As one well studied example, the activation of protein kinase A (PKA) signalling by the second messenger cyclic AMP (cAMP) is precisely restricted to discrete subcellular regions [11]. This is primarily achieved by a diverse set of cytoplasmic scaffolds collectively known as A-kinase anchoring proteins (AKAPs). AKAPs bind to PKA regulatory subunits via a well characterized amphipathic α-helix, localizing them to distinct cellular loci near PKA’s substrates [12]. Compartmentalization of PKA allows its enzymatic activity to be directed in a spatially defined and temporally specified manner and disregulation of this compartmentalization has pathophysiological consequences [13].

Although the E1A proteins from multiple HAdVs can synergize with cAMP to alter viral and cellular gene expression [14–18] the exact mechanism remains unclear. Interestingly, HAdV-12 E1A binds directly to the regulatory subunits of PKA, resulting in the relocalization of one isoform from the cytoplasm to the nucleus [19,20]. These results suggest that E1A may function as a ‘viral AKAP’ by redirecting the subcellular localization of PKA to alter transcription.

Here we show that HAdV E1A mimics cellular AKAPs in both appearance and function. We found that the PKA RIα and RIIα subunits are conserved targets of most HAdV E1A species. Structural modeling and a docking analysis predict a remarkable similarity between the binding of E1A and cellular AKAPs to PKA, which was confirmed experimentally. In addition, we observed E1A-mediated relocalization of PKA subunits and competition between E1A and cellular AKAPs during infection that contribute to HAdV gene expression and overall viral replication. Together, our studies identify E1A as the first known viral AKAP, and reveal a unique example of viral subversion of the PKA pathway via structural mimicry.

## Results

### Multiple PKA subunits are conserved targets of HAdV E1A

**Fig 1:**
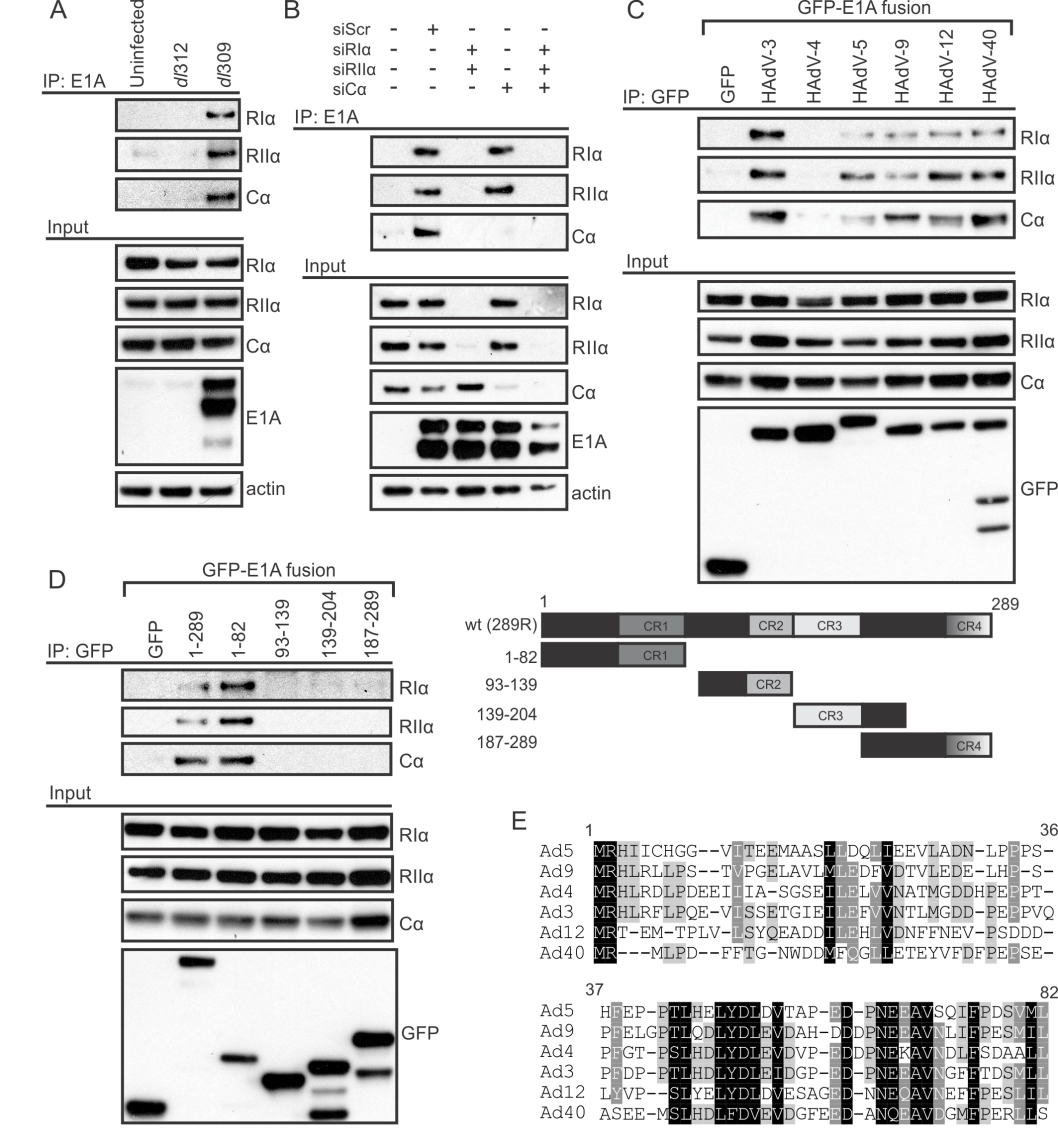
Multiple subunits of PKA are conserved targets of HAdV-5 E1A during infection. A, B) A549 cells were infected with either WT (dl309) or ΔE1A HAdV-5 (dl312) at an MOI of 5 pfu/cell and cell lysates were harvested for co-immunoprecipitation. A) Associations between E1A and endogenous PKA subunits RIα, RIIα, and Cα are shown. B) Cells were treated with the indicated siRNAs (shown in the inset panel) prior to infection. Associations between E1A and endogenous PKA subunits RIα, RIIα, and Cα are shown. The interaction between E1A and the PKA catalytic subunit Cα required the presence of PKA regulatory subunits, indicating an indirect association. E1A’s interaction with the regulatory subunits was unaffected by the Cα knockdown. C, D) HT1080 cells were co-transfected with PKA subunits and various E1A constructs expressed as fusions to EGFP and cell lysates were harvested for co-immunoprecipitation. C) Full-length E1A proteins from 6 different HAdV species all interacted with PKA subunits RIα, RIIα, and Cα to varying degrees, with the exception of HAdV-4. D) Of the E1A fragments tested (shown in the inset panel) only the N-terminus was sufficient for interaction with PKA. This region of E1A has several regions of high amino acid sequence conservation between various HAdV species (E).

The E1A proteins from multiple HAdVs synergize with cAMP to alter viral and cellular gene expression. A direct interaction between HAdV-12 E1A and the type I and type II regulatory subunits of PKA (RIα and RIIα) was previously reported, but has not been investigated further [19]. It was also not known if this interaction was specific to HAdV-12 E1A. To further explore the E1A-PKA interaction, A549 lung adenocarcinoma cells were infected with wildtype (WT) HAdV-5 or a ΔE1A virus and co-immunoprecipitations were performed (Fig 1A). Similarly to HAdV-12 E1A, HAdV-5 E1A interacted with endogenous PKA regulatory subunits RIα and RIIα. Interestingly, we also found a previously unknown interaction between HAdV-5 E1A and the endogenous PKA catalytic subunit Cα. siRNA-mediated downregulation of specific PKA subunits demonstrated that E1A’s association with Cα required expression of RIα and RIIα (Fig 1B). This suggests that the interaction with the Cα subunit may be indirect and that E1A binds the entire PKA holoenzyme.

To determine if the interactions between E1A and the PKA subunits are evolutionarily conserved across the different HAdV species, HT1080 fibrosarcoma cells were transfected with vectors expressing the PKA subunits and the largest E1A isoform from six different HAdV species. Co-immunoprecipitation analysis revealed that RIα, RIIα, and Cα all interacted with each of the E1A proteins tested, with the exception of HAdV-4 (Fig 1C). The conservation of the E1A-PKA interaction across most HAdV species suggests that targeting of PKA is an important evolutionarily conserved function of E1A.

E1A is comprised of a series of protein interaction modules that often can function independently [8]. To grossly define which portion of E1A is required for PKA interaction, lysates from HT1080 cells expressing the PKA subunits and the indicated large fragments of HAdV-5 E1A expressed as EGFP-fusions, were subjected to Co-IP. The N-terminal 82 residues of HAdV-5 E1A were sufficient for association with PKA (Fig 1D). Interestingly, this region of E1A has been previously shown to be involved in alterations in cAMP signalling [21]. In addition, the interaction of HAdV-12 E1A with PKA similarly mapped to residues 1–79 in a yeast interaction assay [19]. As can be seen from the amino acid sequence alignment, there are several areas of high sequence similarity in this region in the E1A proteins from various HAdV species (Fig 1E).

### The sequence of E1A that binds PKA resembles a cellular AKAP

**Fig 2:**
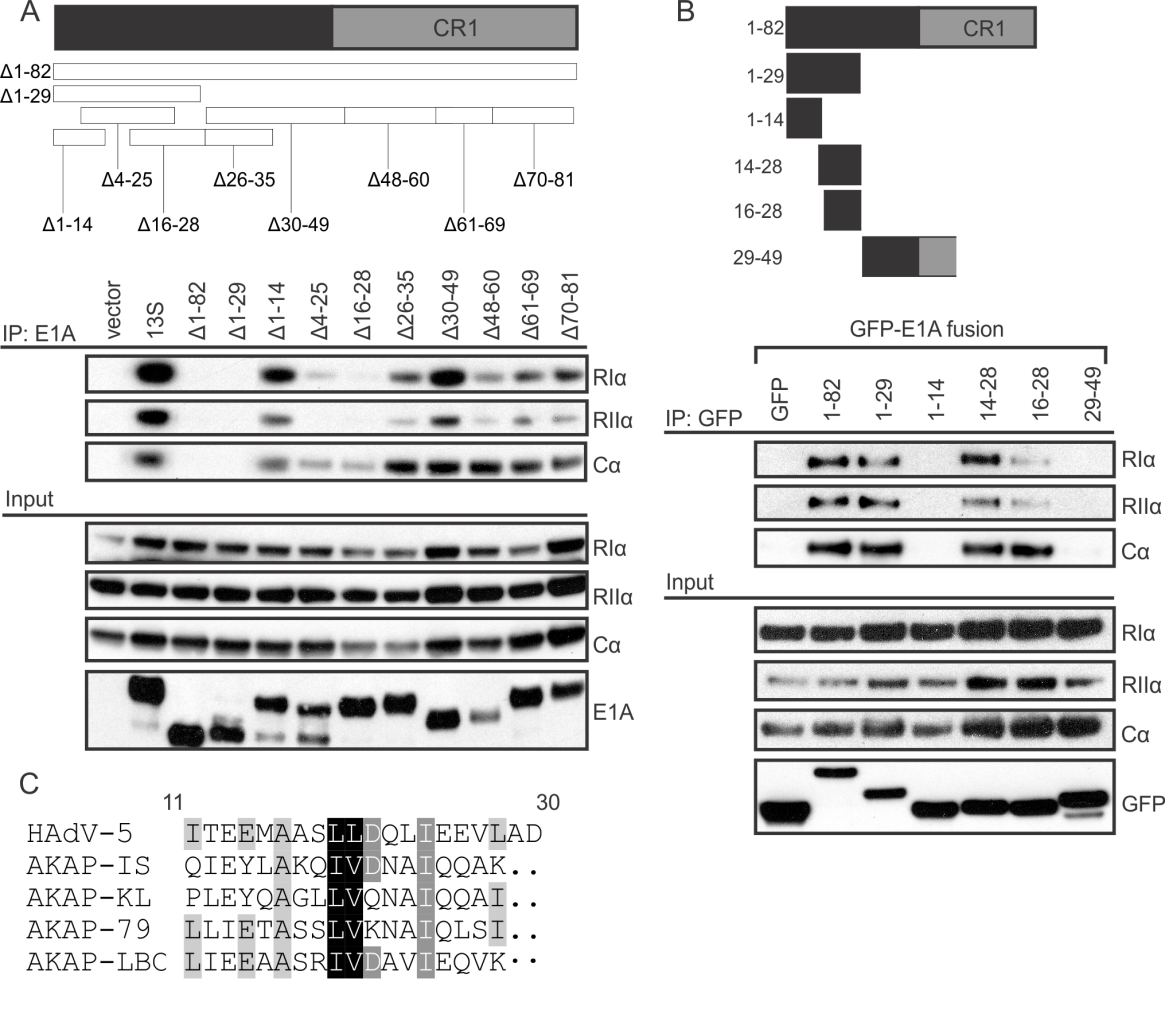
E1A contains an AKAP-like domain that is necessary and sufficient for binding PKA. HT1080 cells were co-transfected with PKA subunits and various E1A constructs and cell lysates were harvested for co-immunoprecipitation. A) Mutational analysis using N-terminal deletion mutants of full-length E1A (shown in the inset panel) revealed that amino acids 14–28 are necessary for binding PKA subunits. B) The N-terminal region of E1A, when expressed as a fragment fused to EGFP (shown in the inset panel), was also sufficient for binding PKA subunits. This region of E1A bears amino acid similarity to a variety of known AKAPs and is predicted to contain an amphipathic α-helix (C).

To determine the minimal region of HAdV-5 E1A necessary and sufficient for PKA interaction, we carried out a detailed mutational analysis of the N-terminus of E1A. Cells were co-transfected with vectors expressing PKA subunits and the indicated E1A mutants, each expressed in the context of full-length HAdV-5 E1A and containing a small in-frame deletion in the N-terminus. As expected, deletion of residues 1–82 abrogated interaction with PKA, confirming that the E1A N-terminus as necessary and sufficient for binding PKA (Fig 2A). Several smaller, overlapping deletions also had similar defects for PKA-binding, specifically Δ1–29, Δ4–25, Δ16–28, and Δ26–35. However, adjacent deletion mutants Δ1–14 and Δ30–49, or more distant deletions retained interaction. This suggests that a region spanning residues 14–29 of HAdV-5 E1A is necessary for PKA binding.

**Fig 3:**
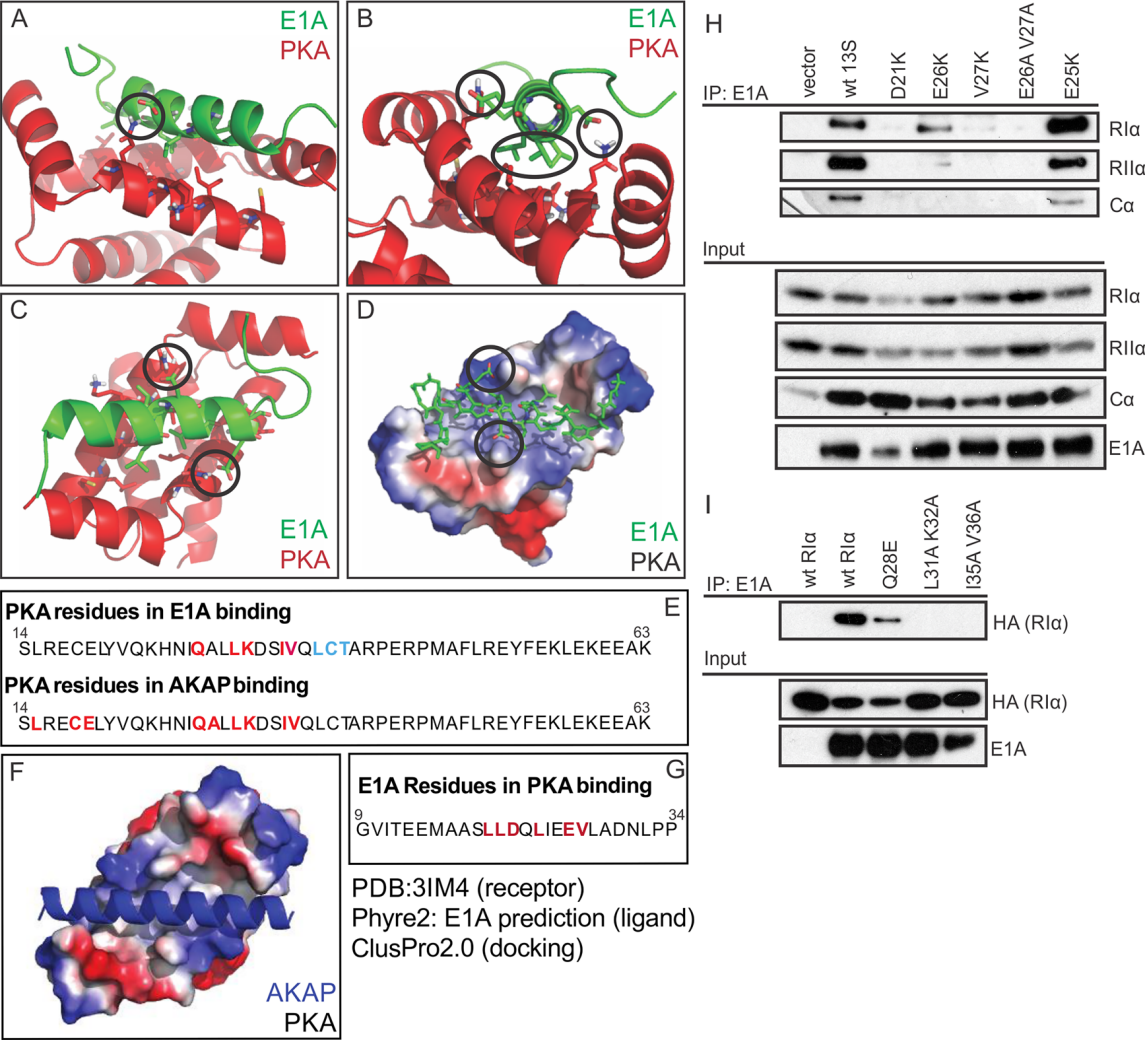
E1A is predicted and confirmed to dock to PKA equivalently to a cellular AKAP. (A,B,C) Cartoon and stick representations of the predicted E1A helix docking to Protein Kinase A regulatory subunit R1α (dimer;red). Residues represented in sticks are within 4A of each other, suggesting potential interactions. Key predicted interactions are circled. (D) Electrostatic surface representation of PKA R1α and alpha carbon tracing of E1A demonstrates limited potential electrostatic interactions, and multiple hydrophobic interactions. (E) Sequence analysis reveals the residues of PKA R1α important for AKAP binding are similar to the residues implicated in the predicted interaction with E1A. (F) Crystal structure of AKAP interacting with PKA regulatory subunit RIα (PDB ID 3IM4). AKAP structure is shown in blue, Surface electrostatics of PKA are depicted, with blue and red representing positive and negative charges respectively. (G) Sequence of the predicted E1A helix and the residues implicated in PKA interaction according to ClusPro2.0 docking. (H) E1A mutants D21K, E26K, V27K and E26A/V27A reduced interaction with RIα, whereas substitution of E25 with K, which is not predicted to alter binding, had no effect. (I) RIα mutants Q28E, L31A K32A and I35A/V36A displayed a reduced ability to bind E1A. See also S1 Fig.

We next co-transfected cells with PKA and small E1A fragments expressed as EFGP fusions. Co-immunoprecipitation on lysates of these cells demonstrates that the 14–28 region of E1A was sufficient to confer an interaction with PKA (Fig 2B). This region is similar in the E1A proteins from most HAdV species (Fig 1E) and also has noticeable sequence similarity to the PKA-binding regions of a number of cellular AKAPs (Fig 2C). Interestingly, AKAPs bind PKA regulatory subunits via an amphipathic α-helix secondary structure motif [22,23], and modeling of the N-terminus of HAdV-5 E1A predicts it also forms an amphipathic α-helix (Fig 3). Furthermore, the E1A proteins from all HAdV species are strongly predicted to form an α-helix in this region [8,21], with the exception of HAdV-4 E1A, which is predicted to form a lower-confidence helix (S1A Fig) and does not bind PKA efficiently (Fig 1C). Taken together, this suggests that E1A binds PKA by structurally mimicking the AKAPs’ amphipathic α-helix motif.

### E1A binds the same surface of PKA targeted by cellular AKAPs

We performed *in silico* molecular modeling to predict the docking of the N-terminus of E1A with PKA. Docking simulations performed using the crystal structure of a dual-specificity cellular AKAP in complex with the RIα homodimer of PKA suggest that the interaction of E1A with RIα is virtually equivalent to that of the cellular AKAP (Fig 3A–3D and 3F). This model predicts a number of distinct interactions between E1A and RIα (Fig 3E and 3G), which were experimentally tested (Fig 3H and 3I). E1A mutants D21K, E26K, V27K and E26A/V27A, reduced the interaction with RIα as predicted, whereas substitution of E25 with K, which is not predicted to alter binding, had no effect (Fig 3H). Similarly, RIα mutants Q28E, L31A K32A and I35A/V36A displayed a reduced ability to bind E1A as predicted by the model (Fig 3I). These results indicate that the docking model can correctly predict key residues necessary for binding, which further suggests that E1A structurally mimics a cellular AKAP in order to bind PKA.

**Fig 4:**
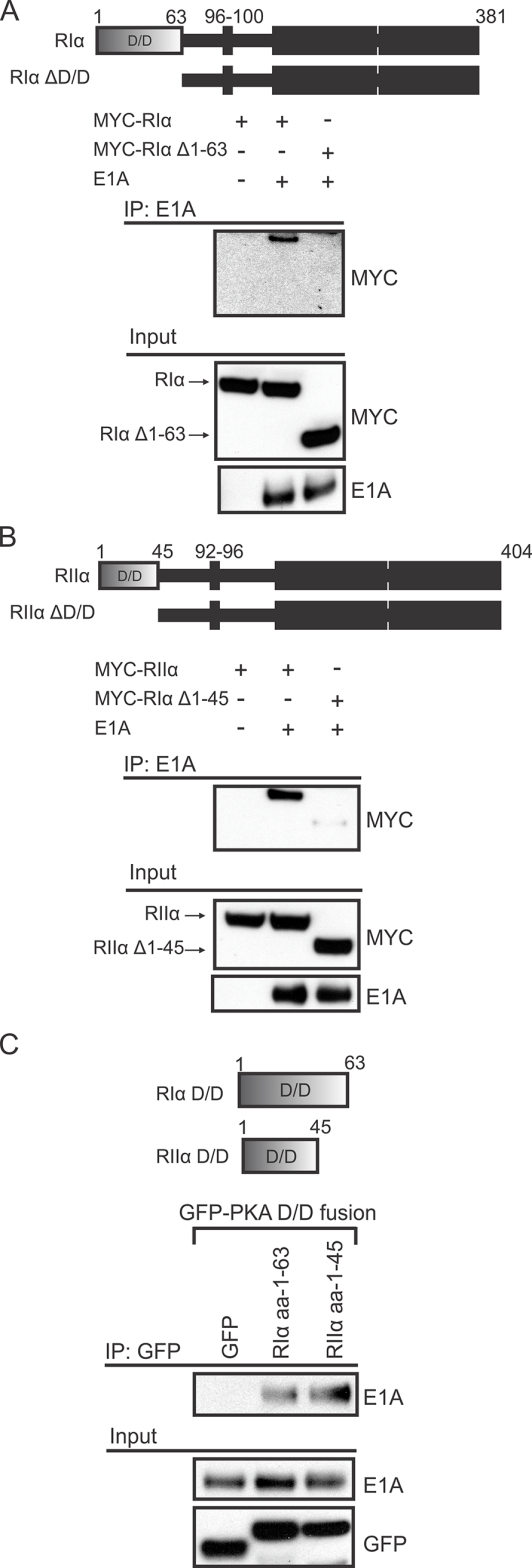
The D/D domains of PKA regulatory subunits are necessary and sufficient for binding E1A. HT1080 cells were co-transfected with E1A and a variety of PKA regulatory subunit constructs and cell lysates were harvested for co-immunoprecipitation. Deletion of the D/D domain (shown in the inset panels) in either RIα (A) or RIIα (B) reduced the interaction with WT E1A. C) When expressed as EGFP fusions (shown in the inset panel) these D/D domains were sufficient for binding E1A. Images are cropped to exclude the IgG heavy chain signal.

Cellular AKAPs bind to the docking/dimerization (D/D) domain located at the N-terminus of the PKA regulatory subunits RIα and RIIα [22,24]. Given the sequence and predicted structural similarity between E1A and cellular AKAPs, we tested if the D/D domain was necessary for the interaction with E1A. Transfected HAdV-5 E1A was unable to co-immunoprecipitate RIα or RIIα lacking their D/D domain (Δ1–63 and Δ1–45, respectively, Fig 4A and 4B). In addition, when the D/D domains of RIα and RIIα were expressed as fusions to EGFP, they alone were sufficient to co-immunoprecipitate E1A (Fig 4C). Thus, the N-terminus of E1A not only resembles an AKAP based on sequence, but also binds to the same site on the PKA regulatory subunits targeted by cellular AKAPs.

### E1A functions as a viral AKAP

**Fig 5:**
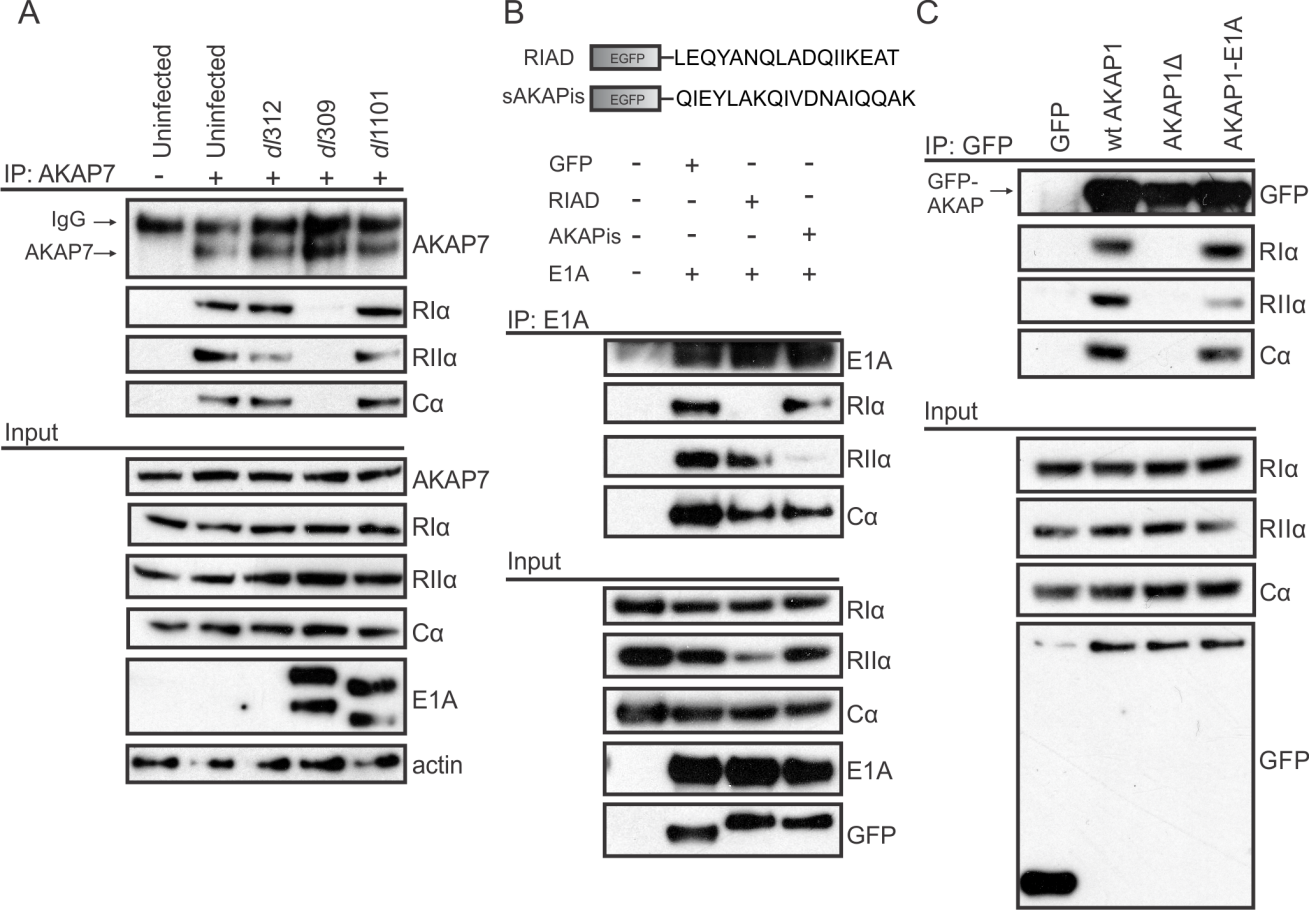
E1A can compete with and function like an AKAP. A) A549 cells were infected with either WT HAdV-5, ΔE1A virus, or a virus that lacks PKA-binding (Δ4–25[dl1101]) and cell lysates were harvested for co-immunoprecipitation. Interactions between the endogenous dual-specificity AKAP7 and PKA subunits was disrupted in the presence of WT E1A, but remained intact in presence of an E1A mutant unable to bind PKA. B) HT1080 cells were co-transfected with E1A, PKA, and the indicated high affinity AKAP-PKA binding inhibitors (shown in the inset panel). The binding inhibitors disrupted the E1A-PKA interactions in an isoform-specific manner. C) HT1080 cells were co-transfected with PKA and the indicated D-AKAP1 construct. An AKAP1 mutant lacking its binding for PKA was rescued by cloning in the AKAP-like sequence of HAdV-5 E1A.

We next determined if the structural similarity between E1A and cellular AKAPs extended to functional similarity. We tested whether E1A could compete with endogenous AKAPs for PKA-binding during infection. A549 cells were infected with WT HAdV-5, a ΔE1A virus, or a virus expressing an E1A mutant unable to bind PKA (Δ4–25). Cell lysates were prepared 18 hours post-infection, subjected to immunoprecipitation with an anti-AKAP7 antibody and any co-precipitating PKA subunits were detected via western blot with specific antibodies for each target. AKAP7 is a dual-specificity AKAP [25], which binds both RIα and RIIα directly, and indirectly binds Cα. Infection with HAdV-5 did not alter the expression of AKAP7 or the various PKA subunits. However, infection disrupted the endogenous interactions between AKAP7 and PKA. Disruption of the AKAP7-PKA interaction during infection required E1A and was dependent on the AKAP like domain in E1A (Fig 5A). These data establish that the AKAP like region in E1A competes with endogenous AKAPs for PKA interaction during infection. These results also suggests that E1A can out-compete at least some cellular AKAPs for binding to PKA, which likely contributes to previously observed perturbation of cellular cAMP signalling by HAdV infection [14,16,21].

We next tested whether *in silico*-designed peptide inhibitors, which block AKAP-PKA interactions by binding PKA regulatory subunits with sub-nanomolar affinities, could affect E1A’s interaction with PKA. These well characterized inhibitors are short peptides expressed as EGFP-fusions which specifically block binding to RIα (RIAD) or RIIα (sAKAPis) [26,27]. HT1080 cells were co-transfected with vectors expressing the PKA subunits, WT E1A, and each of the inhibitors. Lysates were subjected to immunoprecipitation with an anti-E1A antibody and interacting PKA subunits were detected by western blot. As expected based on their high affinity, both RIAD and sAKAPis competitively reduced E1A’s interaction with PKA in a subunit-specific manner, reinforcing E1A’s role as a dual-specificity viral AKAP (Fig 5B).

Using an expression construct for a known cellular dual-specificity AKAP (AKAP1) [27], we next tested E1A’s ability to rescue the PKA-binding function of this AKAP when its PKA-binding domain was deleted. HT1080 cells were co-transfected with PKA subunits and EGFP-fusions of WT AKAP1, an AKAP1 mutant lacking its PKA-binding domain (AKAP1Δ), or an AKAP1 construct with E1A residues 14–28 cloned in lieu of the deletion (AKAP1-E1A). Lysates were subjected to immunoprecipitation with an anti-EGFP antibody and co-precipitating PKA was detected via western blot. As expected, the AKAP1Δ mutant lost the ability to bind PKA. However, incorporation of the E1A AKAP-like sequence into this mutant rescued PKA-binding to WT levels (Fig 5C). Together, these results strongly suggest that the AKAP-like motif in E1A is functionally indistinguishable from that found in an authentic cellular AKAP.

### E1A alters PKA subcellular localization

**Fig 6:**
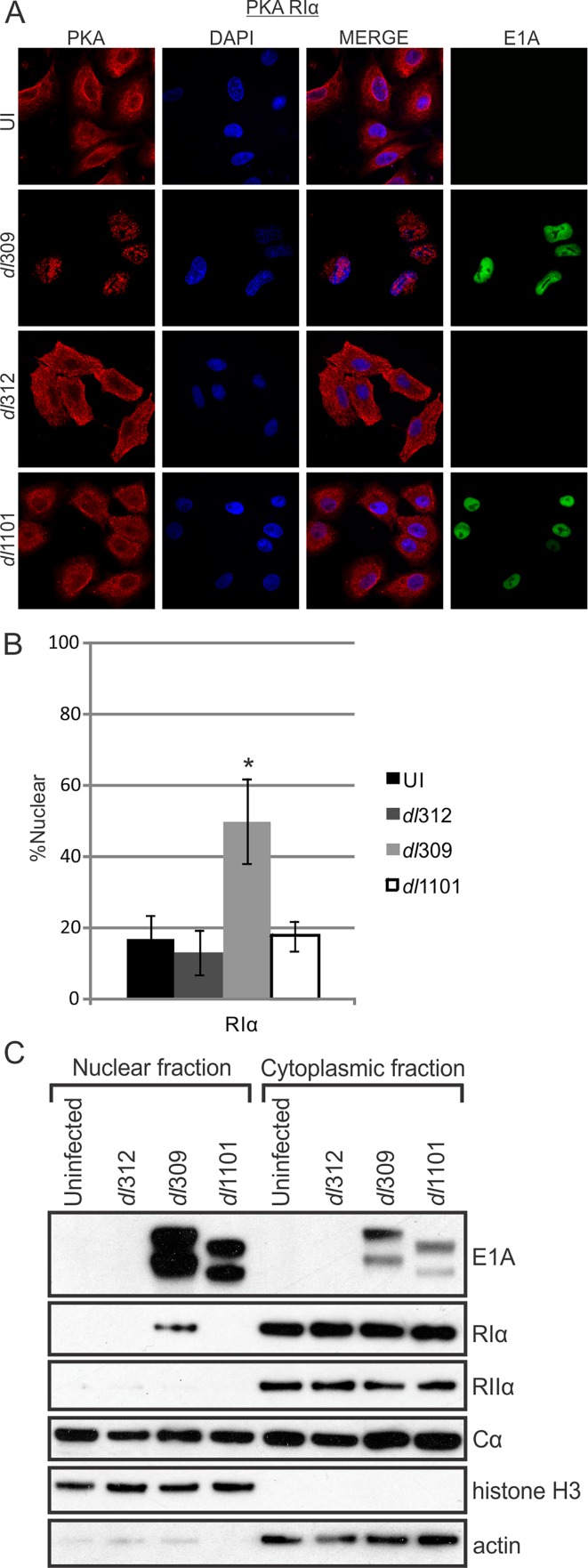
E1A alters PKA R1α subcellular localization during infection. A549 cells were infected with either WT HAdV-5, ΔE1A virus or a virus mutant unable to bind PKA (Δ4–25). Cells were fixed, permeabilized and stained for confocal immunofluorescence. In the presence of WT E1A, RIα exhibited a drastic shift from exclusively cytoplasmic localization into the cell nucleus (A). This redistribution did not occur during ΔE1A or Δ4–25 infections. RIIα and Cα were not re-localized in the same manner as RIα (S2A Fig). B) Quantification of nuclear signal relative to total cellular signal. Statistically significant differences are denoted (*p<0.001) n = 50. C) Nuclear and cytoplasmic extracts from infected cells were prepared by biochemical fractionation and the presence of E1A and PKA subunits in each fraction were detected by western blot. The presence of nuclear RIα in WT-infected cells confirms the results observed by immunofluorescence. See also S2, S3 and S4 Figs.

Transfection of cells with HAdV-12 E1A induces a relocalization of the RIIα subunit of PKA from the cytoplasm to the nucleus [19]. We tested E1A’s ability to alter PKA’s subcellular localization *in vivo* during a HAdV-5 infection (Figs 6 and S2A). A549 cells were infected with WT virus (dl309), a ΔE1A virus (dl312), or the Δ4–25 E1A deletion mutant virus (dl1101) that does not bind PKA. At 18 hours post-infection, cells were subjected to immunofluorescence staining and biochemical fractionation to determine the subcellular localization of PKA. In WT-infected cells, endogenous RIα was rerouted from the cytoplasm into the nucleus. Additionally, in infected cells, RIα appeared to overlap with the HAdV-5 encoded DNA-binding protein (DBP), suggesting possible co-localization with viral replication centres during infection (S3 Fig). In contrast, the distribution of PKA subunits in cells infected with either the ΔE1A or Δ4–25 virus resembled uninfected cells. Thus, the relocalization of RIα is E1A-dependent and requires the AKAP motif. Subcellular localization of RIIα appeared to be unaffected by the presence of E1A and Cα retained its nuclear/cytoplasmic phenotype in both uninfected and infected cells, thereby rendering any conclusions regarding its relocalization difficult (S2A Fig).

Interestingly, RIα, but not RIIα, is similarly trafficked into the nucleus of HEK293 cells, which stably express HAdV-5 E1A. Knockdown of E1A in HEK293 cells reduces the amount of RIα in the nucleus, further suggesting that E1A is functioning as an AKAP in these cells to redistribute PKA (S2B Fig). Additionally, A549 cells transiently transfected with HAdV-5 E1A conferred a similar result, whereas cells transfected with HAdV-4 E1A (which does not bind PKA via Co-IP [Fig 1]) did not affect PKA localization (S4 Fig). These results demonstrate that the AKAP function of HAdV-5 E1A can alter the localization of PKA whereas E1A from a HAdV species that does not bind PKA lacks this biological function. Interestingly, HAdV-5 E1A appears to primarily affect type-I PKA, whereas the previously reported effect of HAdV-12 E1A was restricted to type-II PKA.

### E1A uses PKA to enhance HAdV gene expression

**Fig 7:**
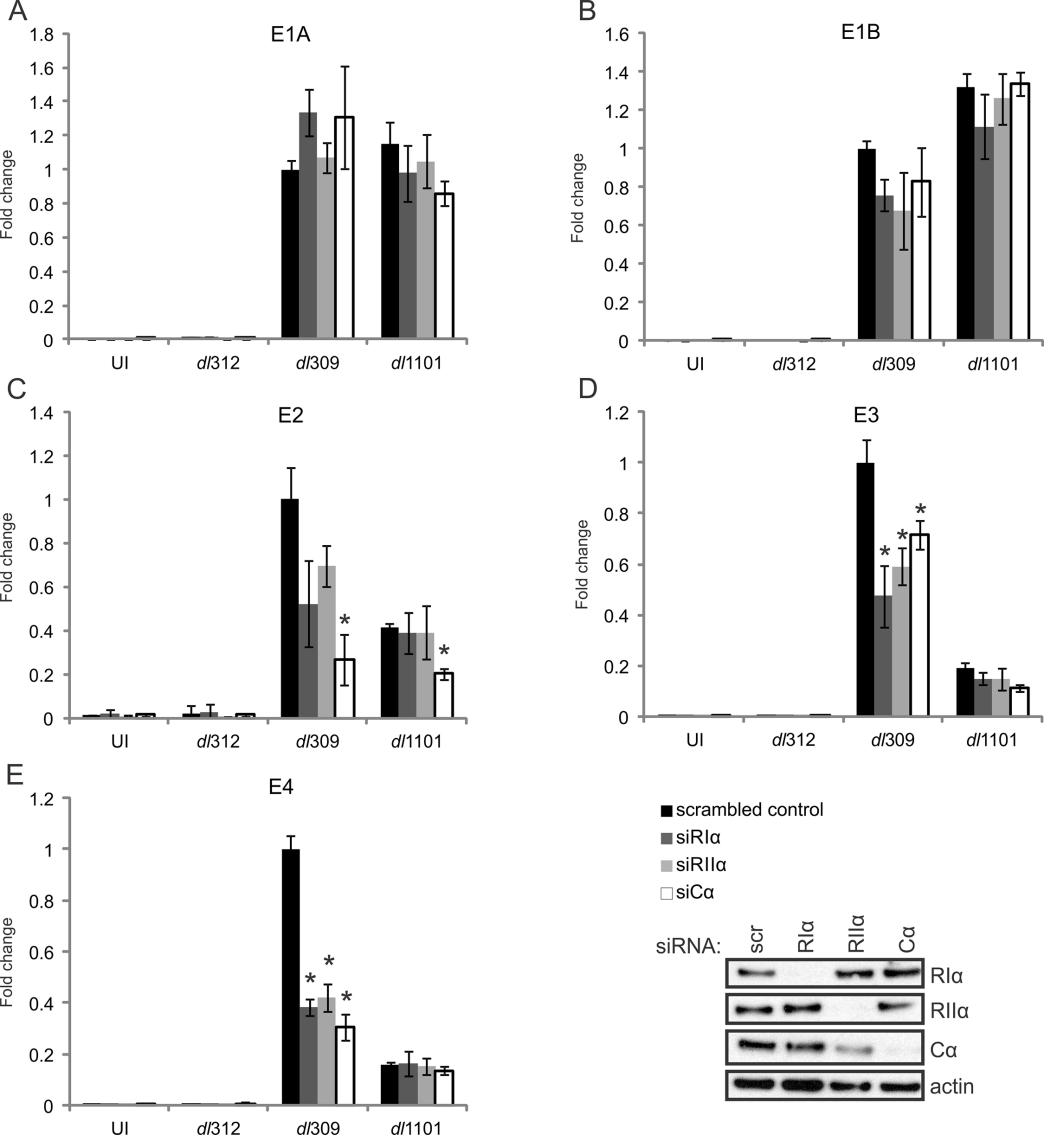
The interaction between E1A and PKA subunits is required for full expression of HAdV-5 E3 and E4 transcripts. A549 cells were treated with control siRNA or siRNA specific for PKA subunits (shown in the inset panel) and infected with either WT HAdV-5 or the indicated mutants. RT-qPCR was performed with a panel of HAdV-5 early genes, normalized to GAPDH and fold change to control treated cells was plotted. Results for the HAdV-5 E1A (A), E1B (B), E2 (C), E3 (D) and E4 (E) transcription units are shown. A statistically significant decrease from control-treated cells is indicated (*p<0.05) n = 3. See also S5 and S6 Figs.

Previous studies indicated that E1A and cAMP synergize to activate viral gene expression [14–16,18,21,28]. To determine if the E1A-PKA interaction contributes to HAdV early gene transcription, A549 cells were first treated with control siRNA or siRNA specific for each PKA subunit and then infected with WT (dl309), ΔE1A (dl312), or Δ4–25 (dl1101) HAdV-5. Cells were harvested 20 hours post-infection, cDNA was prepared and the expression of a panel of HAdV early genes known to be activated by E1A was determined by quantitative real-time PCR. Knockdown of RIα, RIIα, or Cα did not affect expression of the E1A (Fig 7A) or E1B (Fig 7B) transcription units for any of the tested viruses. However, mRNA levels were significantly reduced for both the E3 (Fig 7D) and E4 (Fig 7E) transcription units in WT virus infected cells treated with siRNA for each of the PKA subunits, demonstrating that PKA plays a role in the regulation of these transcription units. Importantly, cells infected with the Δ4–25 virus also showed decreased expression of E3 and E4 as compared to WT infection, and this was not further reduced by knockdown of any PKA subunit (Fig 7D and 7E). This is fully consistent with the inability of this mutant E1A protein to bind PKA and relocalize it to the nucleus. Mechanistially, Chromatin immunoprecipitation (ChIP) experiments showed that PKA’s catalytic subunit (Cα) was recruited to the HAdV E3 and E4 promoter regions in an E1A-dependent manner (S6 Fig). In contrast, E1A did not specifically recruit Cα to the E1B or GAPDH promoters, whose transcription was unaffected by the E1A-PKA interaction (Fig 7). These results strongly support a mechanism of early gene activation that relies on the AKAP function of E1A.

Although knockdown of PKA regulatory subunits had no statistically significant effect on E2 transcripts, knockdown of the catalytic subunit reduced E2 expression for both WT and Δ4–25 virus (Fig 7C). This suggests an independent effect for PKA on this transcription unit that does not rely on the AKAP motif.

To extend the observations that PKA plays a role in HAdV gene expression, we further examined PKA’s role in HAdV-5 protein production (S5 Fig). A549 cells were treated with control siRNA or siRNA specific for each PKA subunits and infected with WT HAdV-5. Cell lysates were collected at 12, 24, and 36 hours post-infection. Viral protein production was assayed by western blot using antibodies against an array of HAdV-5 proteins representing both early and late transcription units. Compared to control-treated cells, knockdown of PKA subunits had no effect on the production of HAdV-5 E1A proteins. In contrast, knockdown of the individual PKA subunits caused a notable reduction in several early proteins. These included a reduction in E3-19K at each time point examined, a reduced level of E4orf6 expression at 24 hours post-infection and a delay in expression of the E2-encoded DBP. E1B-55K was also reduced, most notably in the RIα knockdown. Interestingly, many of the late proteins also exhibited lower expressions levels in PKA-knockdown cells, including hexon, penton, protein V, and protein VII. This confirms a role for PKA in regulating HAdV-5 gene expression.

### E1A uses PKA to enhance viral replication

**Fig 8:**
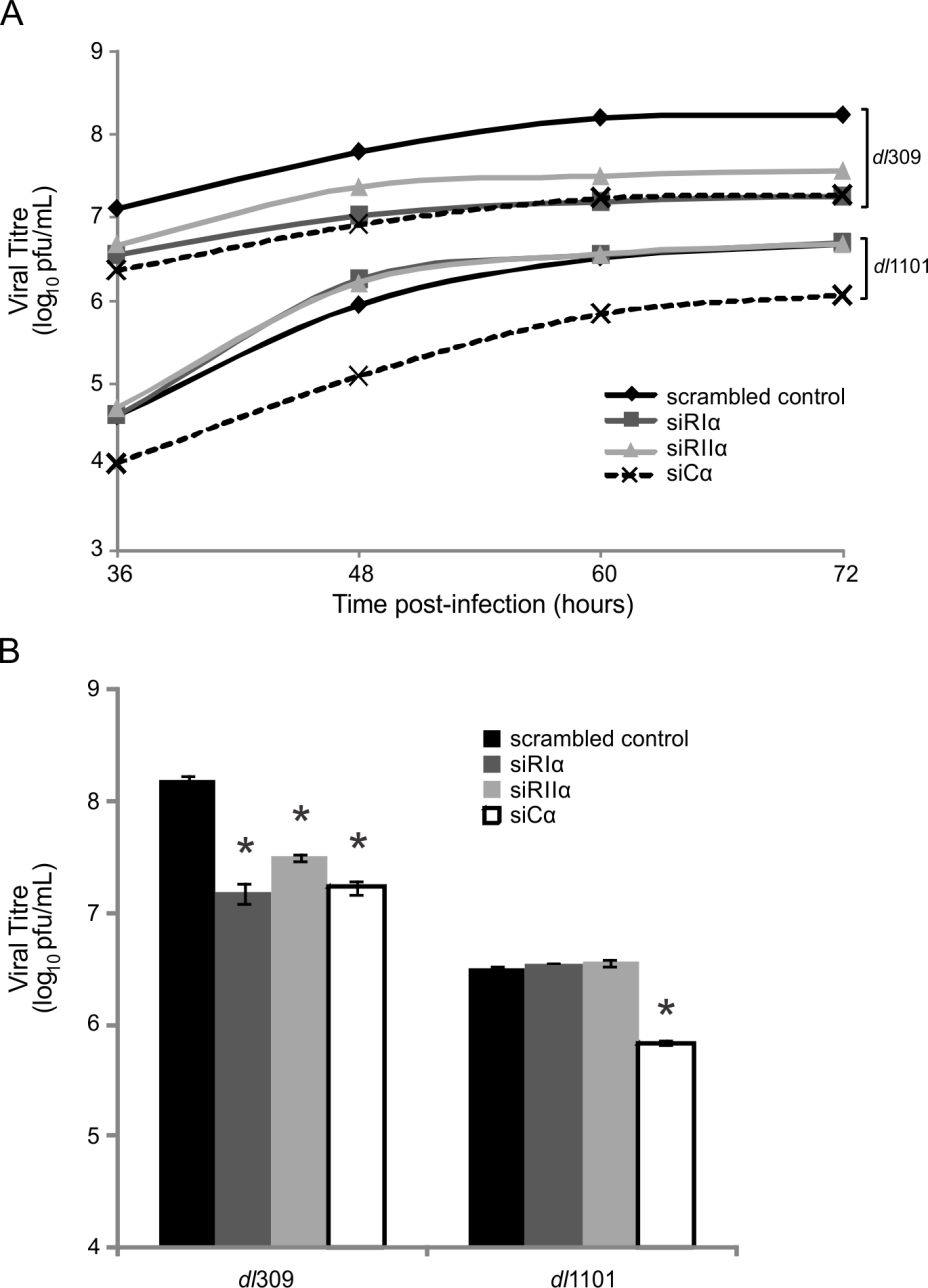
Interactions between E1A and PKA are required for full HAdV-5 progeny production. A549 cells were treated with control siRNA or siRNA specific for PKA subunits and infected with either WT HAdV-5 or a virus encoding E1A unable to bind PKA (Δ4–25). Cells were collected at various time points up to 72 hr post-infection. Production of infectious progeny virus was quantitatively assayed by plaque formation on HEK293 cells (A). Data are shown over 36–72 hr. Growth of WT virus was decreased by knockdown of each PKA subunit. Growth of HAdV E1A Δ4–25 was not affected by knockdown of PKA regulatory subunits, but was affected by knockdown of PKA Cα (though to a lesser extent than WT HAdV-5). (B) Total viral progeny production at 60 hours post-infection when virus replication appeared to peak in most conditions. All values are represented as mean ± SEM. The statistically significant reductions in viral titres compared to control-treated cells are denoted (*p<0.01).

To establish the biological significance of E1A’s role as a viral AKAP, we also assessed the effect of the E1A-PKA interaction on viral replication (Fig 8). A549 cells were treated with either control siRNA or siRNA specific for each PKA subunit and infected with either WT or Δ4–25 HAdV-5. Production of infectious virus progeny was assayed at various time points over 72 hours by plaque assay. The production of WT virus was reduced by knockdown of each PKA subunit when compared to control-treated cells. Although the production of the Δ4–25 virus was reduced as compared with WT infection, it was not further reduced by knockdown of either RIα or RIIα. This again suggests that the lack of PKA-binding by this E1A mutant is functionally equivalent to PKA knockdown. These results indicate that HAdV replication requires PKA activity and that E1A’s interaction with PKA’s regulatory subunits is required for WT-levels of replication. Interestingly, we observed a reduction in progeny production for both WT and Δ4–25 virus in cells treated with Cα-specific siRNA. However, the observed reduction compared to control-siRNA treated cells was more severe in the WT infection, suggesting an additional role for PKA in HAdV-5 infection that is E1A-independent and specific for PKA’s catalytic subunit. Altogether, these results confirm that the targeting of PKA by the AKAP motif in E1A is a critical aspect in the HAdV-5 replicative cycle.

## Discussion

**Fig 9:**
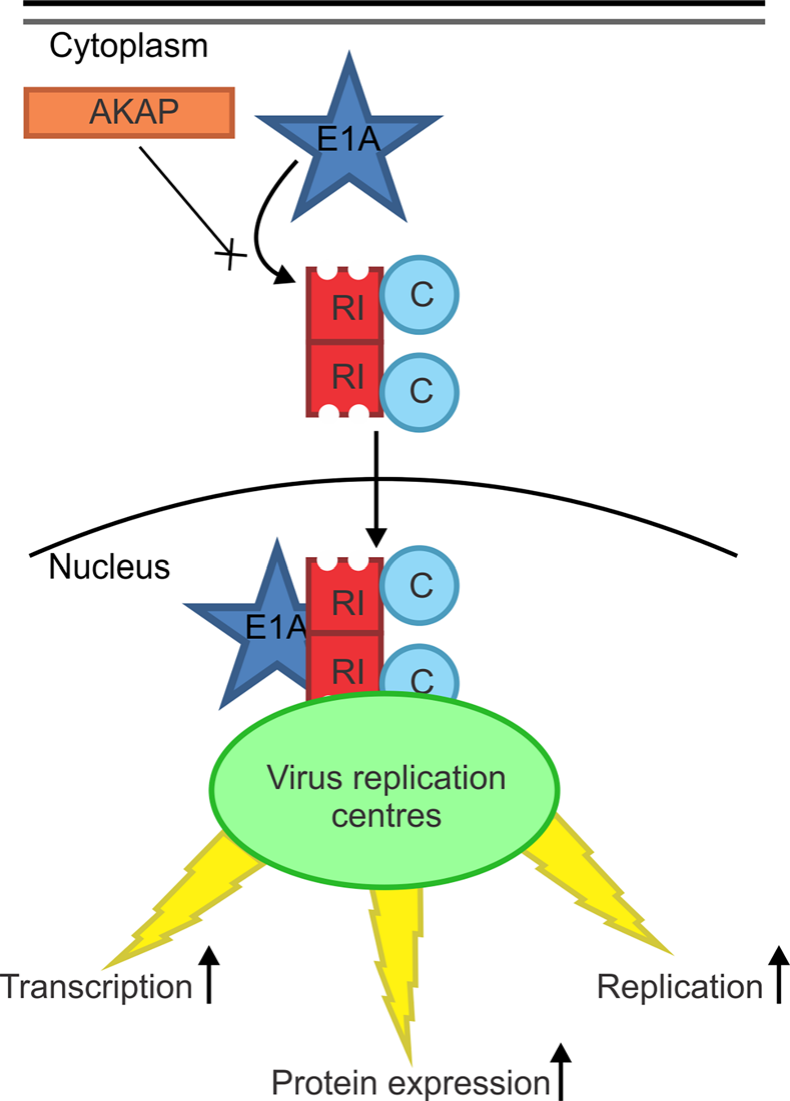
E1A functions as a viral AKAP. During infection, the viral E1A protein interacts with the PKA holoenzyme. This occurs via structural mimicry of the PKA interaction domain of cellular AKAPs. As a consequence, competition occurs between the viral AKAP and cellular AKAPs for interaction with PKA. The interaction of E1A with PKA leads to a relocalization of a subset of PKA to the nucleus, likely to HAdV early gene promoters within virus replication centres. This interaction and relocalization is required for efficient viral transcription, protein expression and progeny production.

Cellular AKAPs function as scaffolds that target PKA and other signaling enzymes to specified subcellular locations. These multivalent anchoring proteins serve as important focal points for the processing and integration of intracellular signalling [29,30]. We report here that the adenovirus E1A oncoproteins function as the first known viral AKAPs. Intriguingly, E1A interacts with the with both the RIα and RIIα subunits of PKA in a way that precisely mimics that of cellular dual-specificity AKAPs. Specifically, we found that E1A bound to the N-terminal D/D domain of the regulatory subunit dimer of PKA, which is the same exact domain targeted by cellular AKAPs [11,12]. We identified a short conserved sequence in HAdV-5 E1A spanning residues 14–28 that was necessary and sufficient for interaction with either RIα or RIIα. Like the PKA interaction domains of cellular AKAPs, this region of E1A is predicted to form an amphipathic α-helix. This apparent structural mimicry allows E1A to bind PKA with an affinity comparable to cellular AKAPs, such that E1A can successfully compete with endogenous cellular AKAPs for PKA interaction during infection (Fig 9).

In support of our *in vivo* and *in vitro* results, molecular modeling based on a known structure of the AKAP/PKA interaction predicts that E1A binds the exact same surface of the PKA regulatory subunit in a fashion virtually identical to that determined for cellular AKAPs (Fig 3). Substitution of specific residues predicted by this model to make contacts reduced the interaction *in vivo*, supporting the validity of this structural model of molecular mimicry.

Functionally, as observed for cellular AKAPs, E1A relocalizes PKA to target sites of action. In the case of E1A, the interaction with PKA induces a specific relocalization to the nucleus, which contributes to viral gene expression and efficient virus propagation during infection. Competition by E1A with cellular AKAPs for PKA interaction may also influence cellular gene expression, which may provide some insight into the previous observations that E1A influences cAMP signalling [14–16,21,28,31]. The E1A region mapped as necessary and sufficient for PKA-binding also overlaps with regions previously implicated in E1A’s ability to act as a transforming oncoprotein [32]. Whether PKA contributes to the transforming ability of E1A remains unknown, though PKA itself has been investigated in a variety of cancer-related functions [13,33,34]

Our results also demonstrate that PKA is a conserved target of the E1A proteins from multiple HAdV species, suggesting that this interaction is functionally important for the virus. The E1A proteins from all HAdV types tested bound PKA strongly, with the exception of HAdV-4 E1A which also failed to relocalize PKA (S4 Fig). Modeling of an interaction between HAdV-4 E1A and PKA predicts that key electrostatic and hydrophobic contacts are absent, which are necessary for the HAdV-5 E1A PKA interaction (Figs S1 and 3H). Interestingly, HAdV-4 is unique as it is the sole member of species E HAdV and arose from an interspecies recombination event between chimpanzee and human adenovirus [35].

As mentioned above, during HAdV-5 infection, E1A was able to out-compete endogenous cellular AKAP7 for PKA interaction; however, there exist a plethora of other, diverse AKAPs with varying affinities for PKA. For example, the *in silico*-designed ‘super AKAPs’ RIAD and sAKAPis [26,27] blocked the binding of E1A to the PKA RIα and RIIα subunits, respectively. Thus, the affinity of the E1A/PKA interaction is not high enough to compete with synthetic AKAPs with sub-nanomolar affinities for PKA. Consequently, these inhibitors are potential tools for further study of E1A function in the context of its role as a viral AKAP.

During HAdV-5 infection, a substantial fraction of the RIα subunit was trafficked from the cytoplasm into the nucleus in an E1A-dependent manner. We also observed signal overlap between RIα and HAdV DBP (S3 Fig), suggesting co-localization with viral replication centres. Interestingly, the HAdV-5 E1A-mediated shift in RIα localization is the opposite finding reported for E1A from HAdV-12, which relocalized RIIα only [19]. While both E1As bound to both type-I and–II PKA in Co-IP assays, our studies suggest that in biologically-relevant conditions they each may exhibit higher affinity or preference for one PKA flavour over another, a property shared by many cellular AKAPs [11,12,27]. The binding affinities and potential preferences of E1A proteins from the other HAdV species during infection remains to be fully explored. It also remains to be determined if type-I and type-II PKA are completely interchangeable, or if there are functional consequences driving the preference of each virus for each regulatory subunit type.

Interestingly, nuclear localization of the PKA holoenzyme is considered relatively unusual, but has been studied in detail in HEK-293 cells [36]. We confirmed nuclear localization of RIα in these cells, which constitutively express HAdV-5 E1A [37]. Our results suggest that nuclear localization of PKA in HEK-293 cells is a likely consequence of the AKAP function of E1A. Furthermore, our data suggests that the results of studies of PKA function in these cells may be confounded by the impact of viral manipulation of this pathway.

The targeting of PKA by E1A is required for maximal expression of the HAdV-5 E3 and E4 transcription units. It appears that E1A is using the regulatory subunits of PKA as a bridge to bind Cα, redistributing it to associate with other E1A binding partners at preferred sites within the nucleus, such as the HAdV early gene promoters (S6 Fig). This could establish new localized connections with cAMP-regulated transcriptional machinery, such as CREB or ATF, at viral or cellular loci. This may help explain the previously-observed ability of E1A to cooperate with cAMP in transcriptional activation [14–16].

The importance of PKA during a productive infection is further underscored by our observation that siRNA-mediated downregulation of PKA subunits reduces progeny production by WT HAdV-5. It is likely that the observed defect in the virus’ ability to express numerous crucial transcripts and proteins in the absence of PKA (or the AKAP function of E1A) contributes greatly to this. It is also possible that the E1A-PKA interaction affects cellular tasks that influence HAdV replication, given that PKA and cAMP have been previously shown to extensively modulate cellular transcription, protein expression, and cell signalling [38–41]. As expected, growth of a virus expressing an E1A mutant unable to bind PKA (Δ4–25) was reduced relative to WT. Importantly, knockdown of regulatory subunits RIα and RIIα did not further reduce the overall replication of this mutant, confirming that the lack of the E1A-PKA interaction contributes to its growth defect. Interestingly, loss of Cα expression negatively affected overall viral replication for both WT and Δ4–25 viruses, suggesting an E1A-independent effect of Cα on the HAdV life cycle. This may be related to reports that PKA activity is involved in dynein-mediated transport of species C HAdV virions to the nucleus during the establishment of infection [42,43].

Although E1A is presently unique in its ability to function as a viral AKAP, the important role of PKA in cellular homeostasis makes it an attractive target for modulation during infection by other viruses. For example, the Herpes simplex virus-1 US3 kinase interacts with and activates PKA to block apoptosis [44]. Varicella-zoster virus also upregulates PKA expression and modulates phosphorylation of PKA substrates to improve replication [45]. More typically, PKA is recruited to phosphorylate viral proteins, altering their stability, folding or ability to interact with other targets [46–49]. As one well characterized example, the E6 oncoprotein from human papillomavirus (HPV) is phosphorylated by PKA during infection, allowing it to interact with numerous cellular proteins [50,51]. While E1A does not appear to be a substrate for PKA, its unique mechanism of commandeering this enzyme via mimicry highlights the diverse ways in which viruses can repurpose the same cellular factors. It is also interesting that rather than encoding an entire PKA ortholog or an entire viral protein to subvert PKA function, HAdV uses a short 15 amino acid fragment of the versatile E1A protein to retask PKA for the benefit of the virus. The fact that the AKAP mimic motif in E1A also overlaps regions required for targeting other cellular regulatory proteins [7,52,53] further demonstrates the incredible effect of selective pressure on maximizing the impact of the relatively limited coding capacity of HAdV.

In summary, we conclusively identify E1A as the first known viral AKAP. We demonstrate that the N-terminus of E1A has evolved to mimic the appearance, structure and function of the PKA interaction domain of cellular AKAPs. Furthermore, we have established that the AKAP function of E1A plays a biologically significant role in redirecting PKA to the nucleus during infection, where it is repurposed to enhance HAdV early gene expression and viral progeny production.

## Materials and Methods

### Cell lines and transfections

Human A549 (provided by Russ Wheeler, Molecular Pathology/Genetics London Health Sciences Centre), HT1080 (purchased from the American Type Culture Collection), and HEK293 cells [37] were grown at 37°C with 5% CO_2_ in DMEM (Multicell) supplemented with 10% fetal bovine serum (Gibco). Plasmids were transfected into A549 and HT1080 cells using XtremegeneHP (Roche) following the manufacturer’s recommendation. After 24 hours of incubation, transfected cells were used for downstream experiments.

### Virus infection of cells

All viruses were derived from the HAdV-5 dl309 background and express the 289R and 243R E1A proteins [54,55]. A549 cells were infected with WT (dl309) or HAdV containing the indicated E1A mutant: ΔE1A (dl312), Δ4–25 (dl1101). Cells were infected at a multiplicity of infection (MOI) of 5 pfu/cell. Cell cultures were infected at 50% confluence and subconfluent cells were collected at indicated time points for downstream experiments.

### RNAi knockdown

Downregulation of PKA subunits RIα, RIIα, and Cα was performed using Silencer Select siRNA (Thermo). Four hours after seeding, siRNA was delivered to A549 cells via transfection with Silentfect (BioRad) according to the manufacturer’s instructions. A scrambled siRNA was used as a negative control. Treated cells were used for experiments 48 hours post-transfection. Downregulation of E1A in HEK293 cells was performed using a cocktail of E1A-specific siRNAs generated by Thermo Fisher’s custom siRNA design platform. All siRNAs used can be found in S1 Table.

### Plasmids

All constructs were expressed in vectors under control of the CMV promoter. WT RIα, RIIα, and Cα were PCR amplified (from Addgene 23741, 23789 and 23495) and cloned into pcDNA4-HA and pCANmyc. RIα Δ1–63 and RIIα Δ1–45 were similarly derived and expressed in pCANmyc. D/D fragments of RIα and RIIα were both expressed as EGFP fusions from pEGFP-N1. E1A fragments were expressed as fusions to EGFP and either described previously (1–82, 93–139, 139–204, 187–289) [56] or derived via PCR and cloned into pEGFP-C2 (1–29, 1–14, 14–28, 16–28, 29–49). WT HAdV-5 E1A and its associated deletion mutants were all expressed in pcDNA3. These constructs were previously described (Δ4–25, Δ26–35, Δ30–49, Δ48–60, Δ61–69, Δ70–81) [56] or generated via PCR (Δ1–82, Δ1–14, Δ1–29, Δ16–28). Point of mutants of E1A (D21K, E6K, V27K, E26A V27A, E5K) and RIα (Q28E, L31A K32A, I35A V36A) were generated by PCR and expressed in pcDNA3 and pcDNA-HA respectively. The largest E1A isoform from the six HAdV species were cloned as EGFP fusions. D-AKAP1 mutants were generated via PCR of a construct kindly provided by Thomas Kuntziger (Oslo) and expressed in pEGFP-C2. RIAD-EGFP and sAKAPis-EGFP were provided by Alan Howe (Vermont).

### Western blotting and co-immunoprecipitation

Cells were lysed in NP40 lysis buffer (150mM NaCl, 50mM Tris-HCL pH 7.5, 0.1% NP-40) with protease inhibitor cocktail. Protein concentrations were determined using BioRad protein assay reagent using BSA as a standard. Immunoprecipitations were carried out at 4°C for 4 hours, or overnight for endogenous interactions. 2% of sample was kept as input control. After washing with NP40 buffer, complexes were boiled in 25 µL of LDS sample buffer for 5 minutes. Samples were separated on NuPage 4–12% Bis-Tris gradient gels (Life Technologies) and transferred onto a PVDF membrane (Amersham). Membranes were blocked in 5% skim milk constituted in TBS with 0.1% Tween-20. All antibodies used can be found in S2 table. Horseradish peroxidise-conjugated secondary antibody was detected using Luminata Forte or Crescendo substrate (Millipore). For biochemical fractionation of infected A549 cells, nuclear and cytoplasmic extracts were acquired using an NE-PER kit from Thermo-Fisher.

### Immunofluorescence microscopy and image analysis

Cells were fixed in 3.7% paraformaldehyde, permeabilized on ice using 0.2% Triton X-100, and blocked using 3% BSA in phosphate-buffered saline (PBS). Samples were incubated in the indicated primary antibody for 1 hour at room temperature or 4°C overnight and another hour at room temperature with secondary antibodies (Alexa Fluor 594 α-rabbit, Alexa Fluor 488 α-mouse) (Life Technologies). Samples were mounted with Prolong Gold reagent containing DAPI (Life Technologies). Confocal images were acquired using a Fluoview 1000 laser scanning confocal microscope (Olympus Corp). Non-confocal images were acquired using an Eclipse Ti-U inverted laser microscope (Nikon). Quantification of total cellular signal and nuclear signal was conducted using ImageJ. Cells were normalized for both cytoplasmic and nuclear size and %nuclear signal was determined as previously described [57].

### Quantitative RT-PCR

Total RNA was prepared with Trizol extraction (Life Technologies). A total of 1 μg of RNA was reverse transcribed into cDNA by random priming using the qScript cDNA supermix (Quanta Biosciences) following the manufacturer’s instructions. Quantification of cDNA was done using Power SYBR-Green mastermix (Applied Biosystems) with oligonucleotide sequences that specifically recognize the indicated target. GAPDH was used as a control for total CDNA along with a no-RT negative control. Results were normalized to the GAPDH and uninfected samples and calculated using the ΔΔCt method [58]. Primers used can be found in S3 Table.

### Chromatin immunoprecipitation

Approximately 10^7^ cells per sample were cross-linked in 2mM ethylene glycol bis(succinimidyl succinate) (EGS) for 1 hour followed by 1% formaldehyde for 15 minutes at room temperature. Reactions were quenched with 0.125M glycine and washed twice with cold PBS. Cell pellets were processed in ChIP buffer 1 (10mM HEPES [pH 6.5], 10mM EDTA, 0.5mM EGTA, 0.25% Triton X-100), ChIP buffer 2 (10mM HEPES [pH 6.5], 1mM EDTA, 0.5 mM EGTA, 200mM NaCl), and ChIP buffer 3 (50mM Tris-HCl [pH 8], 10mM EDTA, 0.5% Triton X-100, 1% SDS, and protease inhibitors). Lysates were sonicated in an ultrasonic bioruptor bath (Diogenode) to yield DNA fragments between 200–500 basepairs. 80 μg of chromatin supernatant was used for ChIP, 1% of this was kept for input controls. Samples were diluted 10-fold in ChIP dilution buffer (50mM Tric-HCl [pH 8], 10mM EDTA, 150mM NaCl, 0.1% Triton X-100, protease inhibitors) and precleared with 30μL of Protein G Dynabeads (Invitrogen) for 1 hour at 4°C. Immunoprecipitations were performed overnight at 4°C using 5μg of the indicated antibody in S2 Table. The next morning, 30μL of Dynabeads were incubated with each sample for 2 hours. Beads were then washed with twice each with wash buffer 1 (20mM Tris-HCl [pH 8], 2mM EDTA, 150mM NaCl, 1% Triton X-100, 0.1% SDS), wash buffer 2 (20mM Tris-HCl [pH 8], 2mM EDTA, 500mM NaCl, 1% Triton X-100, 0.1% SDS), and wash buffer 3 (10mM Tris-HCl [pH 8], 1mM EDTA). Immunocomplexes were extracted twice with 150μL of elution buffer (0.1M NaHCO_3_, 1% SDS). 25μL of 2.5M NaCl was added to the 300μL pooled elutions and incubated overnight at 65°C to de-crosslink the complexes. DNA was purified using a PCR purification kit (Thermo). qPCR using SYBR-Green was performed as described previously using 80nM oligos and 0.5μL of ChIP DNA per 15μL reaction.

### Statistical analysis

All experiments were carried out with three biological replicates performed in duplicate. Graphs represent mean and standard error of the mean (S.E.M.) of all biological replicates. For western blots a representative image was selected. Statistical significance of numerical differences was calculated using one-way ANOVA and Holm-Sidak post-hoc comparison between experimental conditions.

### Docking methods

To model the interaction between PKA and E1A, we first performed a structural prediction of the amino terminus of E1A by submitting the primary sequence to Phyre 2 [59]. The predicted structure of E1A was subsequently docked onto PKA (PDB ID: 3IM4) using the standard settings profile of ClusPro2.0 [60]. Residues forming an E1A-PKA binding interface within 4 Angstroms were selected for further experimental analysis. All images were generated in the PyMOL Molecular Graphics System, Version 1.8 Schrödinger, LLC. Additional *in silico* comparisons of HAdV-5 and HAdV-4 E1A were conducted using Clustal Omega [61] and the UCL Department of Computer Science’s PSI-PRED protein sequence analysis workbench [62].

## Supporting Information

S1 Fig Related to Fig 3: HAdV-4 E1A is not predicted to form a helix that is capable of binding PKA in an equivalent manner as the HAdV-5 E1A AKAP-like sequence. (A) The PSI-PRED protein sequence analysis workbench was used to predict the helical propensity of the N-terminal regions of HAdV-5 and HAdV-4 E1A. Although both sequences are predicted to form helices, HAdV-4 has a lower confidence in forming this secondary structure. (B) When attempting to dock the HAdV-4 E1A sequence to RIα using Clus-Pro, electrostatic interactions at both the amino and carboxy ends of this lower confidence structure are absent that are predicted to contribute to the AKAP like interaction with PKA observed with HAdV-5 E1A. As expected, in the absence of these interactions, even in the most energy minimized states calculated for HAdV-4, HAdV-5 E1A demonstrated a more stable energy minimization. (C) Using Clustal, the sequences of HAdV-5 and HAdV-4 E1A are compared, with the residues demonstrated as crucial for PKA-binding in Fig 3H highlighted. The corresponding residues in HAdV-4 E1A are quite different and lack the requisite chemical properties to form bonds with PKA. Additionally, several bulky alphatic residues present in HAdV-5 E1A, which appear to stabilize the interaction with PKA via hydrophobic interactions, are also absent in HAdV-4 E1A.
(TIF)

S2 Fig Related to Fig 6: Subcellular localization of RIIα and Cα during HAdV-5 infection and localization of all PKA subunits in HEK293 cells. (A) A549 cells were infected with either WT HAdV-5 (dl309), ΔE1A virus (dl312) or a virus lacking PKA-binding (dl1101; Δ4–25). Cells were fixed, permeabilized and stained for confocal immunofluorescence. RIIα appears cytoplasmic in all experimental conditions. Cα appears nuclear-cytoplasmic in all experimental conditions, although it may be enriched for nuclear localization in the presence of WT E1A. (B) HEK293 cells (which are stably transformed due to expression of HAdV-5 E1A and E1B) were stained and individual PKA subunits and were demonstrated to have a similar localization phenotypes as in HAdV-infected cells. The nuclear relocalization of RIα appears more diffuse and less punctate in these cells, possibly due to lack of recruitment to viral replication centres as there is no infection occurring in these virally transformed cells. In a separate experiment, endogenous E1A was successfully knocked down to undetectable levels via siRNA-transfection (the knockdown efficiency of various E1A-specific siRNAs generated for this experiment is shown in the inset panel). The amount of RIα detected in the nucleus is greatly reduced when E1A expression is knocked down. Scale bars represent 200μm.
(TIF)

S3 Fig Related to Fig 6: Co-staining of PKA and viral replication centres during HAdV-5 infection. A549 cells were infected with the indicated virus (MOI 5) and were subsequently fixed, permeabilized and stained with antibodies specific for the indicated PKA subunits or HAdV-5 DNA-binding protein (DBP) and DAPI as indicated. Images were acquired on a Nikon Eclipse inverted laser microscope. During WT infection, a portion of nuclear RIα appears to co-stain with the HAdV-5 DBP, suggesting overlap with viral replication centres. This is not observed during infection with mutant virus encoding E1A incapable of binding PKA (dl1101). Under both conditions, RIIα appears to remain cytoplasmic, whereas Cα maintains a diffuse nuclear/cytoplasmic localization.
(TIF)

S4 Fig Related to Fig 6: HAdV-4 E1A does not relocalize PKA subunits. A549 cells were transfected with EGFP-tagged constructs for full-length HAdV-5 or HAdV-4 E1A. Cells were fixed, permeabilized and stained with antibodies for PKA subunits and DAPI as indicated. Unlike HAdV-5 E1A, HAdV-4 E1A was unable to noticeably relocalize PKA, suggesting that an AKAP like protein-protein interaction between E1A and PKA is required for a shift of a subset of PKA into the nucleus.
(TIF)

S5 Fig Related to Fig 7: PKA is required for WT levels of HAdV-5 protein production. A549 cells were treated with control siRNA or siRNA specific for PKA subunits and infected with WT HAdV-5 (dl309; MOI of 5). Cells were harvested at 12, 24, and 36 hr post-infection and viral protein production was assayed by western blot using antibodies against representative proteins from an array of HAdV-5 transcription units.
(TIF)

S6 Fig Related to Fig 7: PKA is recruited to HAdV early gene promoters in an E1A-dependent manner. A549 cells were infected with the indicated viruses at an MOI of 5 and harvested 20 hours post-infection. Chromatin immunoprecipitation (ChIP) was performed with antibodies specific for the indicated proteins. DNA was probed via qPCR for the presence of multiple HAdV early gene promoters (E1B, E3, and E4) and a cellular GAPDH promoter previously shown to be unaffected by E1A in similar conditions (Fonseca *et al*. 2013). Data was normalized to input samples and compared to a non-specific control antibody and ΔE1A-infected cells. A statistically significant increase from ΔE1A-infected cells for each specific ChIP reaction is indicated (* p<0.05, n = 3). In WT-infected cells (dl309), the catalytic subunit (Cα) is specifically recruited to the HAdV E3 and E4 promoters whose transcription was shown to be affected by the E1A-PKA interaction. This recruitment is E1A-dependent as neither ΔE1A HAdV (dl312) or virus incapable of binding PKA (dl1101; Δ4–25) could recruit Cα. In contrast, Cα is not recruited to the GAPDH promoter and while it was present on the E1B promoter, this was independent of E1A and does not appear to affect transcription (Fig 7). Interestingly, neither regulatory subunit of PKA was directly recruited to the HAdV genome. Instead, it appears E1A uses the interaction with the PKA regulatory subunits to retask the catalytic component of the holoenzyme to sites of action in the nucleus.
(TIF)

S1 Table List of silencing RNAs used in this study. (DOCX)

S2 Table List of antibodies used in this study. (DOCX)

S3 Table List of primers used in this study. (DOCX)
