# Supplementary figures and images for: Functional and Structural Mimicry of Cellular Protein Kinase A Anchoring Proteins by a Viral Oncoprotein
Source: PLoS Pathog. 2016 May 3;12(5):e1005621. doi: 10.1371/journal.ppat.1005621 (PMC4854477; doi:10.1371/journal.ppat.1005621)

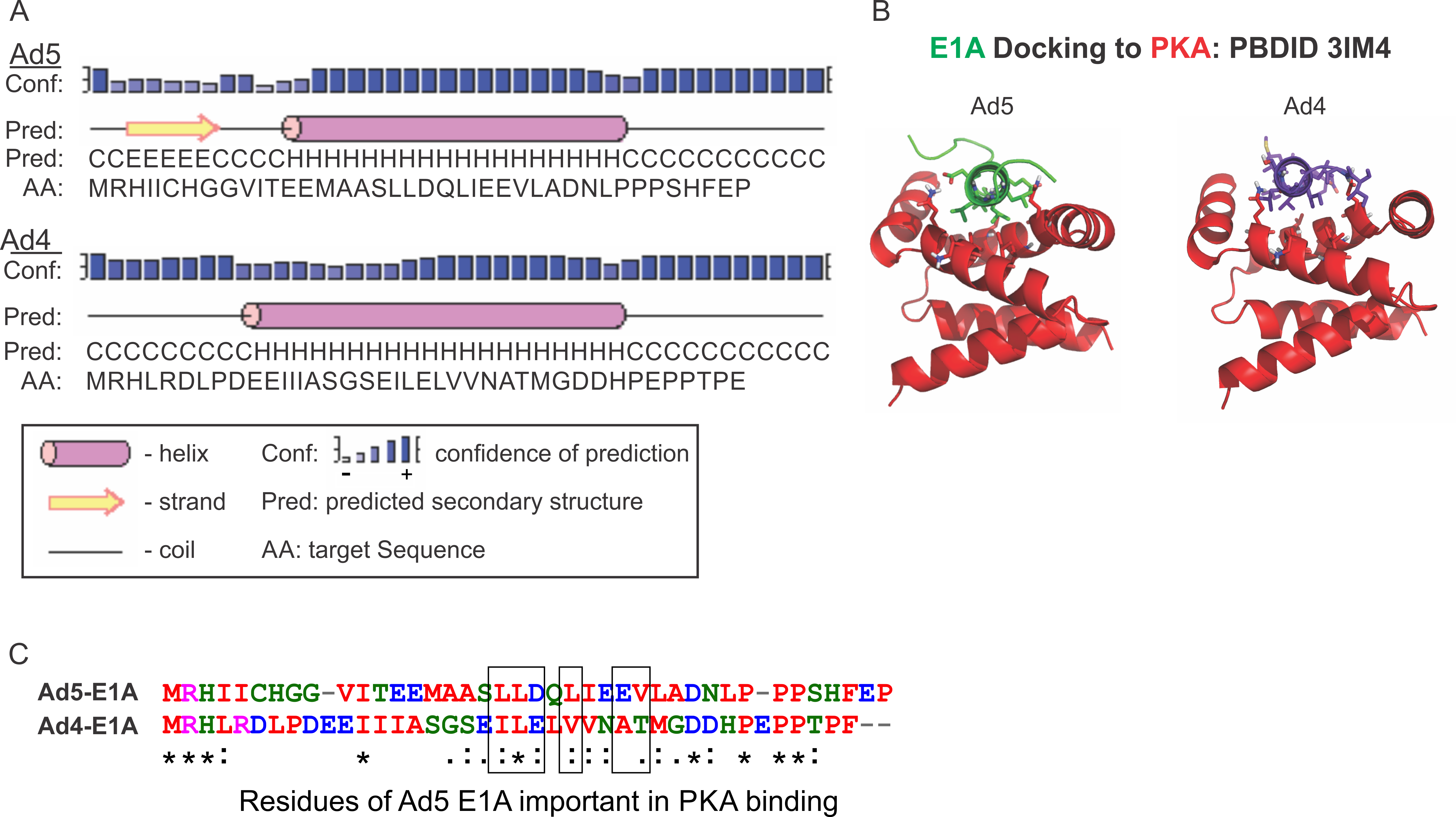

Supplement: S1 Fig — (A) The PSI-PRED protein sequence analysis workbench was used to predict the helical propensity of the N-terminal regions of HAdV-5 and HAdV-4 E1A. Although both sequences are predicted to form helices, HAdV-4 has a lower confidence in forming this secondary structure. (B) When attempting to dock the HAdV-4 E1A sequence to RIα using Clus-Pro, electrostatic interactions at both the amino and carboxy ends of this lower confidence structure are absent that are predicted to contribute to the AKAP like interaction with PKA observed with HAdV-5 E1A. As expected, in the absence of these interactions, even in the most energy minimized states calculated for HAdV-4, HAdV-5 E1A demonstrated a more stable energy minimization. (C) Using Clustal, the sequences of HAdV-5 and HAdV-4 E1A are compared, with the residues demonstrated as crucial for PKA-binding in Fig 3H highlighted. The corresponding residues in HAdV-4 E1A are quite different and lack the requisite chemical properties to form bonds with PKA. Additionally, several bulky alphatic residues present in HAdV-5 E1A, which appear to stabilize the interaction with PKA via hydrophobic interactions, are also absent in HAdV-4 E1A. (TIF) [file ppat.1005621.s001.tif]

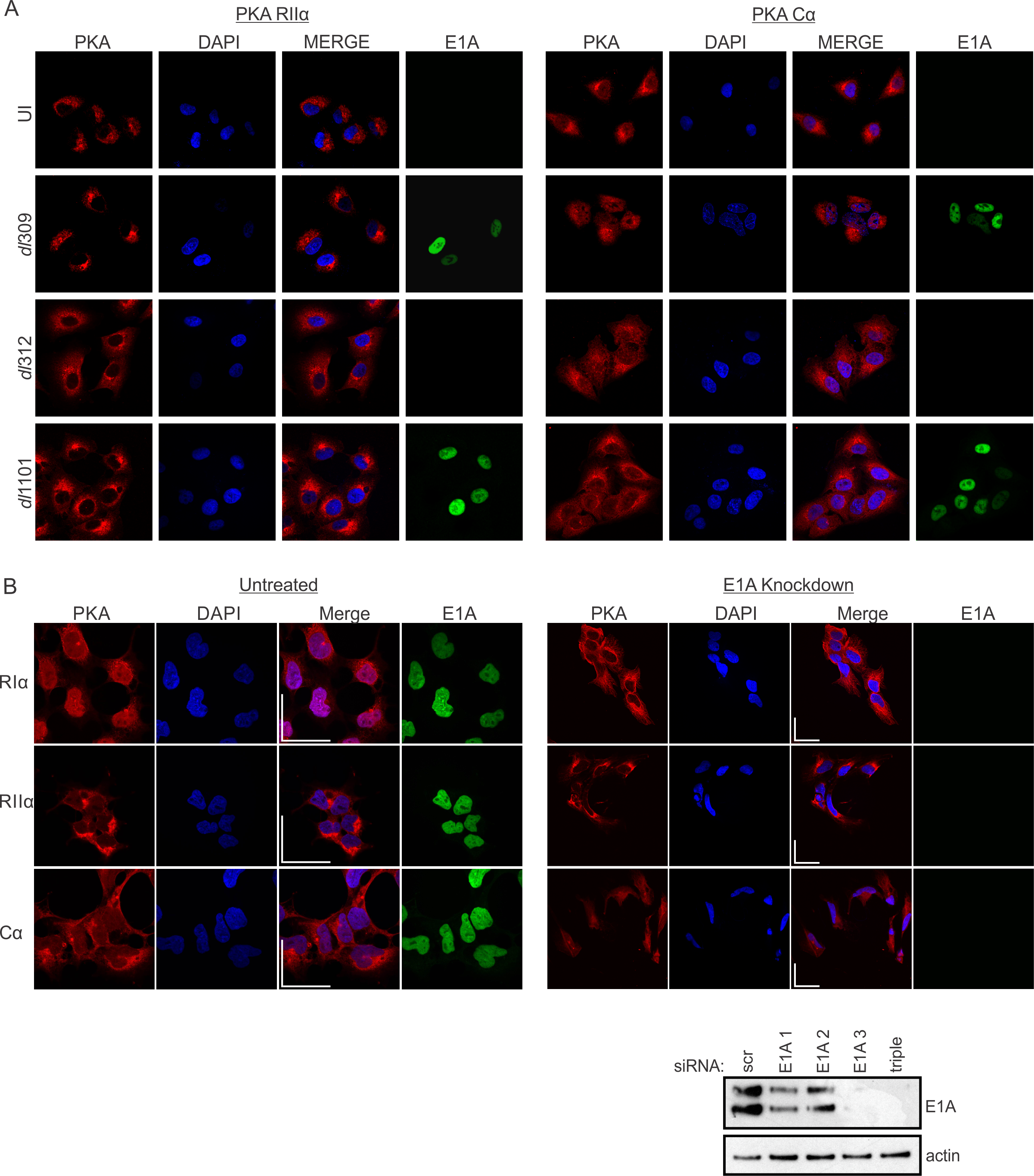

Supplement: S2 Fig — (A) A549 cells were infected with either WT HAdV-5 (dl309), ΔE1A virus (dl312) or a virus lacking PKA-binding (dl1101; Δ4–25). Cells were fixed, permeabilized and stained for confocal immunofluorescence. RIIα appears cytoplasmic in all experimental conditions. Cα appears nuclear-cytoplasmic in all experimental conditions, although it may be enriched for nuclear localization in the presence of WT E1A. (B) HEK293 cells (which are stably transformed due to expression of HAdV-5 E1A and E1B) were stained and individual PKA subunits and were demonstrated to have a similar localization phenotypes as in HAdV-infected cells. The nuclear relocalization of RIα appears more diffuse and less punctate in these cells, possibly due to lack of recruitment to viral replication centres as there is no infection occurring in these virally transformed cells. In a separate experiment, endogenous E1A was successfully knocked down to undetectable levels via siRNA-transfection (the knockdown efficiency of various E1A-specific siRNAs generated for this experiment is shown in the inset panel). The amount of RIα detected in the nucleus is greatly reduced when E1A expression is knocked down. Scale bars represent 200μm. (TIF) [file ppat.1005621.s002.tif]

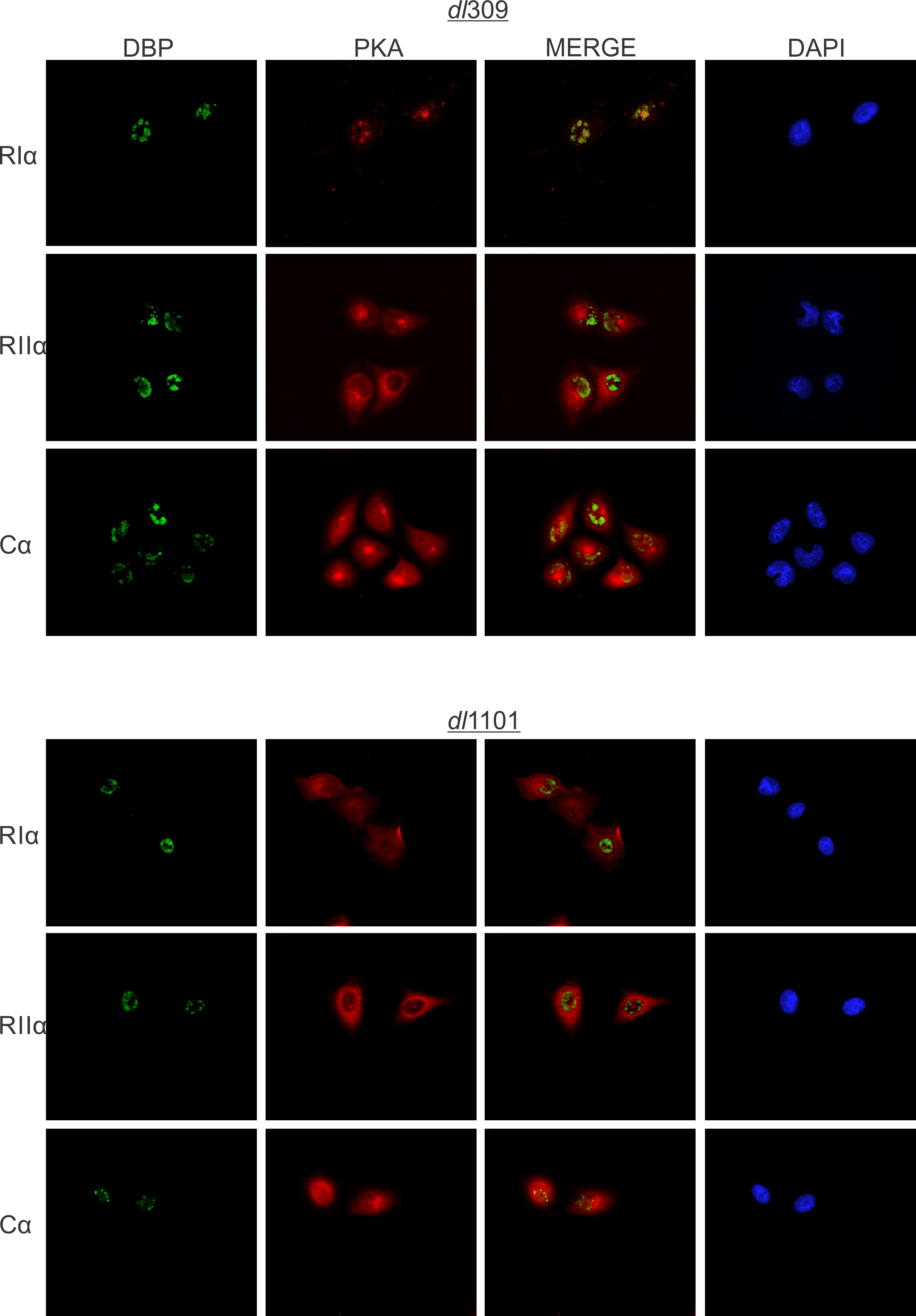

Supplement: S3 Fig — A549 cells were infected with the indicated virus (MOI 5) and were subsequently fixed, permeabilized and stained with antibodies specific for the indicated PKA subunits or HAdV-5 DNA-binding protein (DBP) and DAPI as indicated. Images were acquired on a Nikon Eclipse inverted laser microscope. During WT infection, a portion of nuclear RIα appears to co-stain with the HAdV-5 DBP, suggesting overlap with viral replication centres. This is not observed during infection with mutant virus encoding E1A incapable of binding PKA (dl1101). Under both conditions, RIIα appears to remain cytoplasmic, whereas Cα maintains a diffuse nuclear/cytoplasmic localization. (TIF) [file ppat.1005621.s003.tif]

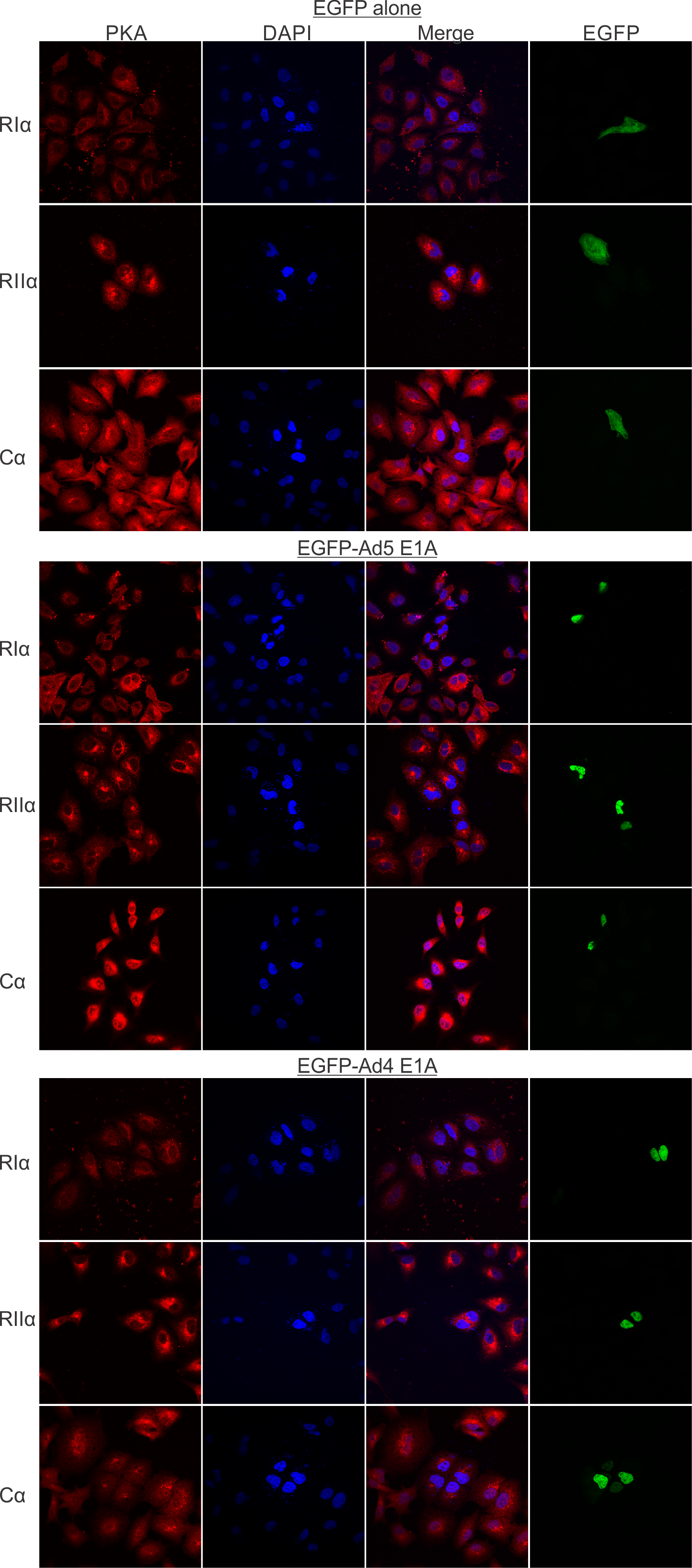

Supplement: S4 Fig — A549 cells were transfected with EGFP-tagged constructs for full-length HAdV-5 or HAdV-4 E1A. Cells were fixed, permeabilized and stained with antibodies for PKA subunits and DAPI as indicated. Unlike HAdV-5 E1A, HAdV-4 E1A was unable to noticeably relocalize PKA, suggesting that an AKAP like protein-protein interaction between E1A and PKA is required for a shift of a subset of PKA into the nucleus. (TIF) [file ppat.1005621.s004.tif]

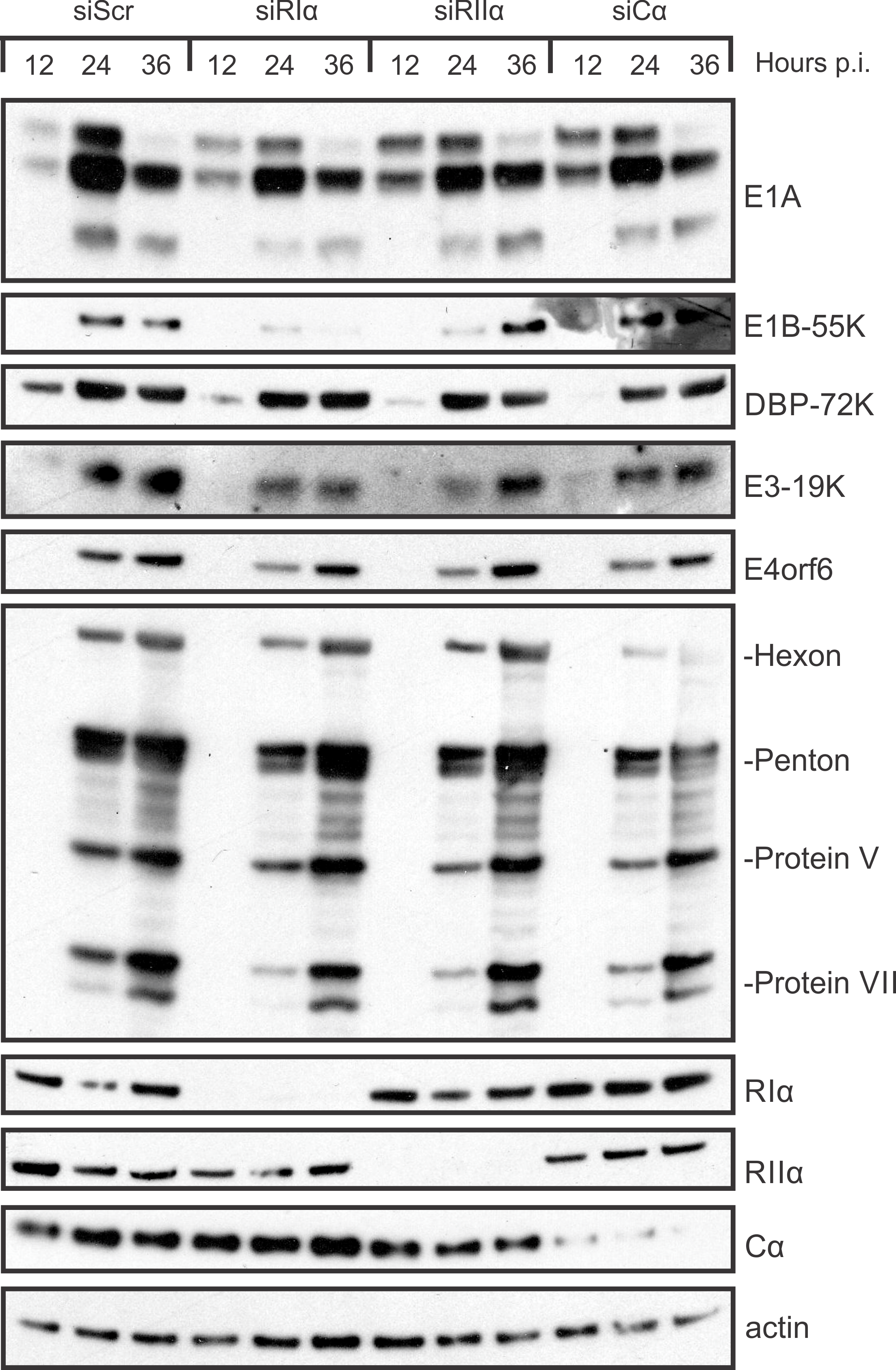

Supplement: S5 Fig — A549 cells were treated with control siRNA or siRNA specific for PKA subunits and infected with WT HAdV-5 (dl309; MOI of 5). Cells were harvested at 12, 24, and 36 hr post-infection and viral protein production was assayed by western blot using antibodies against representative proteins from an array of HAdV-5 transcription units. (TIF) [file ppat.1005621.s005.tif]

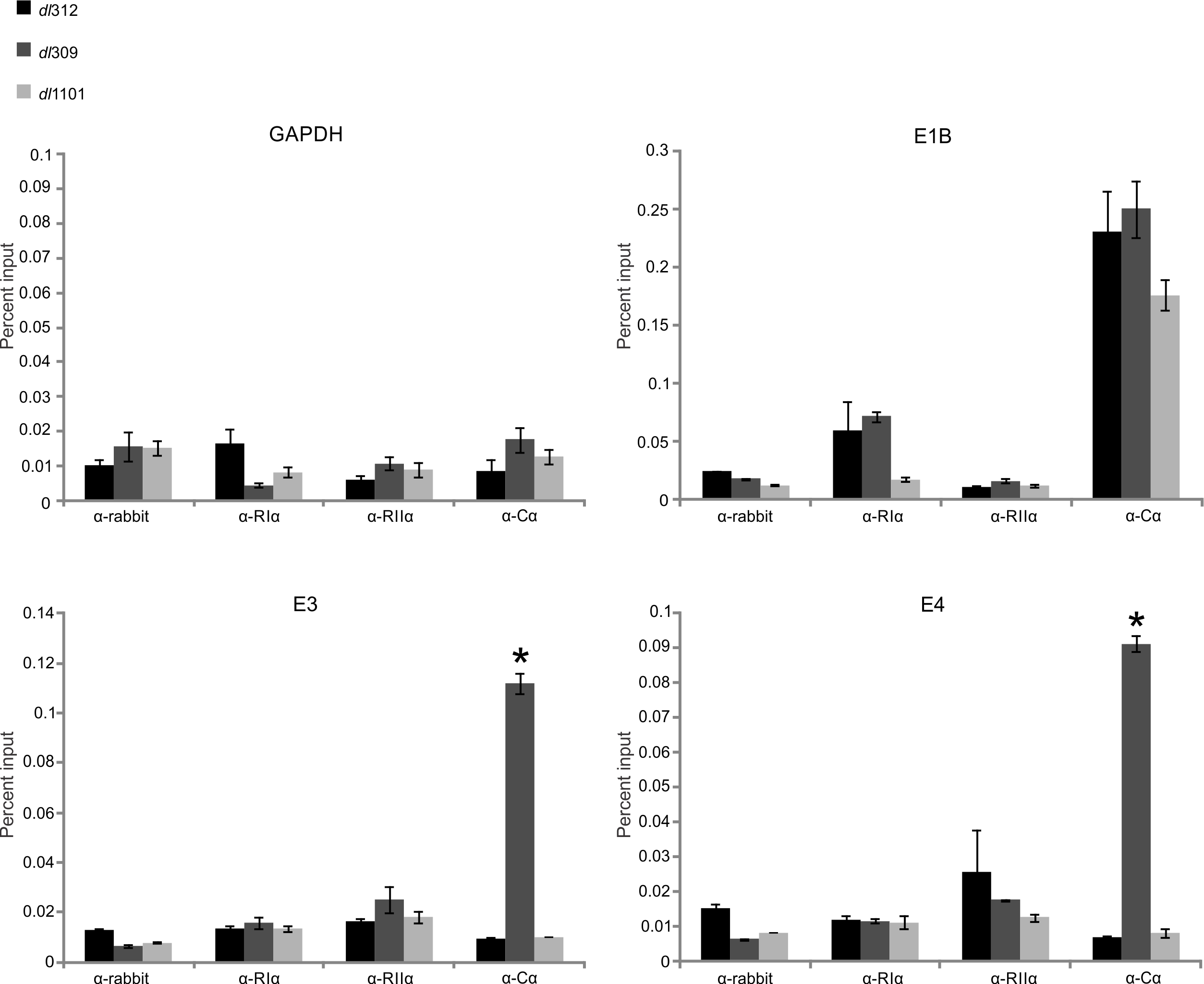

Supplement: S6 Fig — A549 cells were infected with the indicated viruses at an MOI of 5 and harvested 20 hours post-infection. Chromatin immunoprecipitation (ChIP) was performed with antibodies specific for the indicated proteins. DNA was probed via qPCR for the presence of multiple HAdV early gene promoters (E1B, E3, and E4) and a cellular GAPDH promoter previously shown to be unaffected by E1A in similar conditions (Fonseca et al. 2013). Data was normalized to input samples and compared to a non-specific control antibody and ΔE1A-infected cells. A statistically significant increase from ΔE1A-infected cells for each specific ChIP reaction is indicated (* p<0.05, n = 3). In WT-infected cells (dl309), the catalytic subunit (Cα) is specifically recruited to the HAdV E3 and E4 promoters whose transcription was shown to be affected by the E1A-PKA interaction. This recruitment is E1A-dependent as neither ΔE1A HAdV (dl312) or virus incapable of binding PKA (dl1101; Δ4–25) could recruit Cα. In contrast, Cα is not recruited to the GAPDH promoter and while it was present on the E1B promoter, this was independent of E1A and does not appear to affect transcription (Fig 7). Interestingly, neither regulatory subunit of PKA was directly recruited to the HAdV genome. Instead, it appears E1A uses the interaction with the PKA regulatory subunits to retask the catalytic component of the holoenzyme to sites of action in the nucleus. (TIF) [file ppat.1005621.s006.tif]
