# Supplementary material for: Functional and Structural Mimicry of Cellular Protein Kinase A Anchoring Proteins by a Viral Oncoprotein
Source: PLoS Pathog. 2016 May 3;12(5):e1005621. doi: 10.1371/journal.ppat.1005621 (PMC4854477; doi:10.1371/journal.ppat.1005621)
Supplement: S1 Table — (DOCX) [file ppat.1005621.s007.docx]

**Table S1. List of silencing RNAs used in this study**

| Gene Target | ThermoFisher ID# |
| --- | --- |
| PRKAR1A | s286 |
| PRKAR2A | s11086 |
| PRKACA | s11065 |
| E1A#1 | Custom(GAUUUUUCCCGAGUCUGUAtt) |
| E1A#2 | Custom(CGGAGGUGUUAUUACCGAAtt) |
| E1A#3 | Custom(CUGUAUGAUUUAGACGUGAtt) |
