# Supplementary material for: Functional and Structural Mimicry of Cellular Protein Kinase A Anchoring Proteins by a Viral Oncoprotein
Source: PLoS Pathog. 2016 May 3;12(5):e1005621. doi: 10.1371/journal.ppat.1005621 (PMC4854477; doi:10.1371/journal.ppat.1005621)
Supplement: S2 Table — (DOCX) [file ppat.1005621.s008.docx]

**Table S2. List of antibodies used in this study**

| Reactivity | Purpose | Description | Company |
| --- | --- | --- | --- |
| RIα | Western | Mouse monoclonal | BD |
| RIα | IF, ChIP | Rabbit polyclonal | Thermo |
| RIIα | Western | Mouse monoclonal | BD |
| RIIα | IF, ChIP | Rabbit polyclonal | Santa Cruz |
| Cα | Western | Mouse monoclonal | BD |
| Cα | IF, ChIP | Rabbit polyclonal | Santa Cruz |
| M73 (E1A) | Western, IP, IF | Mouse monoclonal | In-house |
| M58 (E1A) | Western | Mouse monoclonal | In-house |
| Actin | Western | Rabbit polyclonal | Sigma |
| EGFP | Western, IP | Rabbit polyclonal | Clontech |
| 9E10 (MYC) | Western | Mouse monoclonal | In-house |
| 3F10 (HA) | Western | Rat monoclonal | Roche |
| AKAP7 | Western, IP | Rabbit polyclonal | Santa Cruz |
| 2A6 (E1B-55K) | Western | Mouse monoclonal | In-house |
| B68 (DBP) | Western, IF | Mouse monoclonal | In-house |
| E3-19K | Western | Rabbit serum | In-house |
| 1807-4 (E4orf6) | Western | Rabbit polyclonal | In-house |
| Ad5 capsid | Western | Rabbit polyclonal | Abcam |
| Histone H3 | Western | Rabbit polyclonal | Abcam |
