# Supplementary material for: Functional and Structural Mimicry of Cellular Protein Kinase A Anchoring Proteins by a Viral Oncoprotein
Source: PLoS Pathog. 2016 May 3;12(5):e1005621. doi: 10.1371/journal.ppat.1005621 (PMC4854477; doi:10.1371/journal.ppat.1005621)
Supplement: S3 Table — (DOCX) [file ppat.1005621.s009.docx]

**Table S3. List of primers used in this study**

| Target | Region | Location | Forward | Reverse | Size |
| --- | --- | --- | --- | --- | --- |
| GAPDH | Transcript | 566-644 | ACTGCTTAGCACCCCTGGCCAA | ATGGCATGGACTGTGGTCATGAGTC | 79 |
| E1A | Transcript | 715-834 | ACACCTCCTGAGATACACCC | TTATTGCCCAGGCTCGTTAAGC | 120 |
| E1B | Transcript | 578-697 | GACAATTACAGAGGATGGGC | CACTCAGGACGGTGTCTGG | 120 |
| E2 | Transcript | 17-155 | GGGGGTGGTTTCGCGCTGCTCC | GCGGATGAGGCGGCGTATCGAG | 138 |
| E3 | Transcript | 13-162 | GAGGCAGAGCAACTGCGCC | GCTCTCCCTGGGCGGTAAGCCGG | 150 |
| E4 | Transcript | 3-129 | GCCCCCATAGGAGGTATAAC | GGCTGCCGCTGTGGAAGCGC | 127 |
| GAPDH | Promoter | 231 bp upstream of TSS | TTCGCCCCAGGCTGGATGG | AGGCGGAGGACAGGATGGC | 126 |
| E1B | Promoter | 1546-1708 | GGTGTAAACCTGTGATTGCG | CAGATGTAACCAAGATTAGCCC | 162 |
| E3 | Promoter | 27501-27633 | GGCGGCTTTCGTCACAGGG | TCTGAAATGTCCCGTCCGG | 132 |
| E4 | Promoter | 35585-35694 | CAGCTCAATCAGTCACAGTGTAAAAAAGGGCC | TGCGGTTTTCTGGGTGTTTT | 110 |
